# Targeting c‐Myc enhances immunotherapy efficacy in combination with Ras inhibitor in triple‐negative breast cancer

**DOI:** 10.1002/ctm2.70484

**Published:** 2025-09-29

**Authors:** Xiaojie Liang, Yiqiu Liu, Ye Zhu, Yuhan Zhao, Fan Ye, Fangyan Gao, Yaqin Shi, Xiaoxiang Guan

**Affiliations:** ^1^ Department of Oncology The First Affiliated Hospital of Nanjing Medical University Nanjing Jiangsu China; ^2^ Department of Oncology The First Affiliated Hospital of Soochow University Suzhou Jiangsu China; ^3^ Jiangsu Key Lab of Cancer Biomarkers Prevention and Treatment Collaborative Innovation Center for Cancer Personalized Medicine Nanjing Medical University Nanjing Jiangsu China

**Keywords:** c‐Myc, immunotherapy, Minichromosome Maintenance Complex Component 2 (MCM2), Salirasib, triple‐negative breast cancer (TNBC), tumour microenvironment (TME)

## Abstract

**Background:**

Triple‐negative breast cancer (TNBC), which lacks hormone receptors and HER2 expression, presents substantial therapeutic challenges in breast cancer treatment. The efficacy of immunotherapy frequently suffers from the immunosuppressive nature of tumour microenvironment (TME). Hence, discovering effective targets to hinder TNBC progression and bolster immunotherapy's effectiveness is paramount. Previous research from our team indicated notable upregulation of c‐Myc in TNBC, and suppressing c‐Myc enhances the efficacy of PD‐L1 blockade in murine models; nevertheless, the precise mechanisms underlying this phenomenon remain elusive.

**Methods:**

We analysed c‐Myc expression and implemented a systematic drug library screening strategy alongside c‐Myc knockdown to pinpoint potential synergistic agents in TNBC cells. To decipher the regulatory mechanisms of this synergy on cellular malignancy, we conducted cell cycle analysis, cell interaction assays, and RNA‐sequencing. Additionally, we established orthotopic and lung metastasis murine models assess how combination therapy influences PD‐L1 blockade efficacy.

**Results:**

Elevated c‐Myc was observed in TNBC and the Ras inhibitor Salirasib was identified as a potent synergistic agent from cell cycle drug library in c‐Myc‐overexpressing TNBC. The application of Salirasib combined with c‐Myc knockdown markedly suppressed tumour cell aggressiveness and induced apoptosis in vitro. Mechanistically, RNA sequencing revealed that the combination therapy blocked MCM2‐mediated DNA replication in TNBC cells, causing G1/S phase arrest and enhancing tumour suppression. In vivo, the combination significantly improved PD‐L1 blockade efficacy, leading to reduction of tumour volume, inhibition of lung metastases, and remodelling of the immune microenvironment in murine TNBC models.

**Conclusions:**

In summary, our investigation identifies a molecular vulnerability in c‐Myc‐driven TNBC, where Ras inhibition reinforces c‐Myc‐targeted therapy and potentiates immune checkpoint blockade, presenting a promising strategy to improve immunotherapy efficacy in TNBC.

## Graphical abstract

1. Ras inhibition (Salirasib) combined with c‐Myc targeting blocks MCM2‐driven DNA replication, inducing cell‐cycle arrest and apoptosis.

2. Dual inhibition remodels the immunosuppressive TME, polarising macrophages to M1 and enhancing PD‐L1 blockade efficacy.

3. This mechanism‐driven immunotherapeutic approach effectively suppresses tumour growth and metastasis in aggressive TNBC.

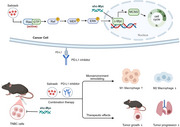

## 1 BACKGROUND

Triple‐negative breast cancer (TNBC), defined by lacking oestrogen receptor (ER), progesterone receptor (PR), and HER2 (Human Epidermal Growth Factor Receptor 2) expression, represents the most aggressive subtype of breast cancer. Accounting for 15%–20% of all breast cancer cases, TNBC contributes significantly to breast cancer‐related mortality worldwide.^1^, ^2^ The heterogeneity of TNBC and the lack of effective therapeutic targets further complicate treatment options.^3^ Thus, chemotherapy remains the primary approach for TNBC treatment. Immunotherapy was considered as a viable treatment approach in several recent clinical trials; however, the results have been mixed and often unsatisfactory. For instance, the KEYNOTE‐012 trial reported an overall response rate (ORR) of 18.5% with pembrolizumab in patients with advanced TNBC,^4^ while the KEYNOTE‐086 trial showed a significantly lower ORR of only 5.7%.^5^ Conversely, the IMpassion130 trial demonstrated a notable improvement in the ORR, reaching 56%, with the combination of atezolizumab and chemotherapy.^6^ These trials underscore the considerable therapeutic potential of immune checkpoint inhibitors (ICIs) but also highlight their variable and limited efficacy in TNBC, emphasising the need to further elucidate the tumour microenvironment (TME) of TNBC and identify effective targets to boost ICI synergy. The above insights justify exploring novel therapeutic strategies to enhance ICI responsiveness.

Multiple studies, including our own prior work, have demonstrated that c‐Myc is markedly elevated expressed in TNBC compared with other breast cancer subtypes.^7^ This aberrant expression plays a pivotal role in driving tumour heterogeneity and stemness, as well as shaping the tumour‐immune landscape within TME.^8^, ^9^, ^10^ Specifically, c‐Myc has been shown to mediate immune infiltration by regulating the plasticity and stem‐like properties of TNBC cells.^10^, ^11^ Given these critical functions, c‐Myc represents a promising therapeutic target for improving ICI efficacy in TNBC. Up to date, a range of molecular strategies – including xenic nucleic acids (XNA), nanoparticles, and RNA‐based therapies – have been employed to inhibit c‐Myc‐driven oncogenic pathways.^12^, ^13^, ^14^ Targeting c‐Myc could effectively curb TNBC progression while potentially expand the population of patients who benefit from immunotherapy. One major mechanism underlying this synergy involves c‐Myc‐mediated regulation of the cell cycle. Our previous work revealed that c‐Myc influences the efficacy of cyclin‐dependent kinase (CDK) inhibitors through modulation of the SOX9–FOXC1 transcriptional axis.^8^ In addition, c‐Myc modulates response to palbociclib, a CDK4/6 inhibitor, via the miR‐29b‐3p/CDK6 signalling pathway.^15^ Collectively, these findings underscore the intrinsic link between c‐Myc signalling, cell cycle control, and the synergistic potential of combining c‐Myc inhibition with immunotherapy. By targeting c‐Myc, novel therapeutic approaches may simultaneously inhibit tumour progression and reprogram the immune microenvironment, thereby enhancing ICI responsiveness and broadening clinical benefit in patients with TNBC. Aberrant Ras pathway activation triggers the epithelial‐to‐mesenchymal transition (EMT), serving an essential function in the propagation and metastasis of breast cancer.^16^, ^17^ Clinicopathological analysis of breast tumour specimens have demonstrated that Ras pathway biomarkers are closely associated with tumour heterogeneity, breast cancer cell proliferation, and long‐term patient outcomes.^18^ Due to its oncogenic role in various cancer types, Ras‐targeted inhibitors are being extensively researched.^19^

In our study, we investigated the synergistic efficacy of c‐Myc inhibition, aiming to devise a combinatorial approach that boosts the effectiveness of immunotherapy in TNBC. Specifically, we explored the potential of targeting Ras‐related pathways in conjunction with c‐Myc inhibition in TNBC. Initiating with a compound library screening, we identified Salirasib as a promising small‐molecule inhibitor that synergises with c‐Myc knockdown in TNBC cell lines. Utilising high‐throughput sequencing, we aimed to clarify the molecular mechanisms that account for the synergistic efficacy, discovering that MCM2‐associated cell cycle arrest is a crucial biological mechanism impeding TNBC proliferation. Furthermore, our findings demonstrated that this combinatorial regimen promoted immune cell infiltration and augmented the efficacy of immunotherapy in TNBC murine models.

## 2 METHODS

### 2.1 Cell lines and culture conditions

The TNBC cell lines MDA‐MB‐231, Hs578T, and Py8119 were obtained from American Type Culture Collection (ATCC, USA). MDA‐MB‐231 and Hs578T were maintained using DMEM medium (Gibco, USA), while Py8119 was maintained in F‐12K medium (Gibco, USA). Both culture media were supplemented with 10% fetal bovine serum (FBS) and 1% penicillin‐streptomycin. All cells were incubated at 37°C in a humidified atmosphere with 5% CO_2。_

### 2.2 Lentiviral transfection and stable cell line construction

Two shRNAs targeting c‐Myc (GGAAACGACGAGAACAGTTGA for Homo sapiens and TGGAGATGATGACCGAGTTAC for Mus musculus) and a non‐targeting control (shControl) were cloned into the LV16 (U6/Luciferase17&Puro) lentiviral vector (GenePharma). Lentivirus production was carried out by introducing shuttle and packaging plasmids into HEK293T cells via RNAi‐Mate (G04001, GenePharma). For lentiviral transduction, TNBC cells were cultured with virus‐containing medium and 5 µg/mL polybrene (H9268, Sigma) at 37°C for 24 h. Stable knockdown cells were selected with puromycin for 4 days, and c‐Myc silencing was confirmed by mRNA and protein analyses 48–72 h post‐infection.

### 2.3 Cell counting kit 8 (CCK‐8) assay

A CCK‐8 kit (HY‐K0301, MedChemExpress) was employed to quantify cell viability. In brief, cells (2000 per well) were dispensed into 96‐well plates and permitted to fully attach prior to further treatment. After 24‐h culture, the cells were treated with different agents from drug library and cultured for another 48 h. Then, the CCK‐8 reagent (10 µL) was added to each well, and the absorbance was measured at a wavelength of 450 nm using a microplate reader (Tecan, Switzerland) after incubation at 37°C for 1 h.

### 2.4 5‐Ethynyl‐2′‐deoxyuridine (EdU) cell proliferation assay

The EdU assay (C0071, Beyotime, China) was performed to quantify cell proliferation, following the recommended procedure. After transduction with shControl or shc‐Myc, TNBC cells (2 × 10⁵ per well) were cultured in 24‐well plates and treated with 10 µmol/L Salirasib for 48 h. Following a 2‐h incubation with 10 µmol/L EdU, cells underwent fixation with 4% paraformaldehyde, permeabilisation with.3% Triton X‐100, and subsequent staining using Azide Alexa Fluor 488. Nuclei were counterstained with Hoechst 33342, and images were captured by an Olympus fluorescence microscope (Olympus, Japan).

### 2.5 Synthetic lethality drug screening

A small molecule inhibitor library comprising 121 cell cycle inhibitors was obtained from Selleck Chemicals (#L5100, Selleck, China). Salirasib was purchased from MedChemExpress (MA, USA). TNBC cells were infected with lentivirus carrying either shRNA targeting c‐Myc or a control shRNA (shControl), followed by treatment with a drug library agent (2 µmol/L), respectively. TNBC cells were transduced with lentiviruses expressing either c‐Myc‐targeting shRNA or control shRNA (shControl), and subsequently exposed to each agent from the drug library at a concentration of 2 µmol/L. After 48 h of treatment, 10 µL of CCK‐8 solution was added to each well, followed by incubation at 37°C for 1 h. The absorbance was then recorded at 450 nm. The cell viability ratio of shControl and shc‐Myc (shControl/shc‐Myc) was determined using CCK‐8 assay, and drugs that caused this ratio value exceeding 1.5 were considered to exhibit synergistic effects when combined with targeting c‐Myc and screened.

### 2.6 Cell invasion assays

Transwell chambers (#3422, Corning, USA) in 24‐well plates, precoated with Matrigel (C0371, Beyotime, China), were utilised to evaluate cell invasion. Upper chambers were seeded with 1 × 10^5^ TNBC cells in serum‐free medium (200 µL), while lower chambers contained complete medium with 10% FBS (600 µL). After 24–48 h at 37°C, membrane‐invaded cells were fixed with 4% paraformaldehyde (BL539A, Biosharp, China) and stained with.1% crystal violet (C0121, Beyotime, China). The invaded cells were subsequently visualised and quantified under an Olympus inverted microscope.

### 2.7 Wound healing assay

Cells were cultured until they achieved complete and uniform adhesion. A linear scratch was then introduced at the centre of each well with a mini sterile pipette tip. Following this, the culture medium was replaced with serum‐free medium. After 24 h of incubation, the extent of cell migration along the wound area was assessed by measuring the percentage of wound closure.

### 2.8 Colony formation assay

Long‐term cell survival was assessed by performing colony formation assays. Briefly, 1000 cells were seeded per well of 6‐well plates for overnight adhesion. Cells were subsequently incubated with compounds from the drug library for 48 h. After a 14‐day incubation, colonies were PBS‐washed, fixed in 4% paraformaldehyde (BL539A, Biosharp, China), and.1% crystal violet‐stained (C0121, Beyotime, China). Colony quantification and statistical analysis followed.

### 2.9 Western blotting assay

The cells were lysed in the lysis buffer for Western blotting and immunoprecipitation (P0013, Beyotime, China), and then they were fully detached using sterile cell scrapers. Protein quantification used an enhanced BCA Assay Kit (P0010, Beyotime, China). Samples underwent separation on 4%–20% SDS‐PAGE precast gels (GenScript, China), then electrophoretic transfer to PVDF membranes. Membranes received 30‐min blocking with QuickBlock™ Protein‐Free Western Blocking Buffer (P0240, Beyotime). Membranes were subsequently incubated overnight with primary antibodies at 4°C, followed by 1‐h RT incubation with corresponding secondary antibodies. Signals were then detected via chemiluminescence. The primary antibodies of anti‐BCL2 (1:5000, #80313‐1‐RR), anti‐BAX (1:5000, #60267‐1‐Ig), anti‐E‐cadherin (1:5000, #80541‐5‐RR), anti‐MMP9 (1:1000, #27306‐1‐AP), and anti‐Vimentin (1:20000, #80232‐1‐RR) were purchased from Proteintech (China). The primary antibodies of anti‐MMP2 (1:1000, A19080), anti‐Ras (1:1000, A19779), anti‐Raf (1:1000, A15033), anti‐p‐Raf (1:1000, AP0012), anti‐MEK1/MEK2 (1:5000, A4868), anti‐p‐MEK1/MEK2 (1:2000, AP1349), anti‐ERK1/2 (1:1000, A4782) and anti‐p‐ERK1/2 (1:1000, AP0974) were obtained from ABclonal (China). The primary antibodies of anti‐c‐Myc (1:1000, #5605), anti‐CyclinD1 (1:1000, #2978), anti‐CDK4 (1:1000, #12790), anti‐CDK6 (1:1000, #13331), anti‐Rb (1:1000, #9313), anti‐p‐Rb (1:1000, #9301), and anti‐MCM2 (1:1000, #3619) were acquired from Cell Signaling Technology (CST, USA). And anti‐GAPDH (1:1000, AF2823) was obtained from Beyotime (China).

### 2.10 Real‐time quantitative PCR (RT‐qPCR) assay

Total RNA was extracted using the FastPure Cell Total RNA Isolation Kit (RC112, Vazyme, China) and reverse‐transcribed into cDNA with the HiScript III RT SuperMix for qPCR (+gDNA wiper) (R323, Vazyme). RT‐qPCR was conducted with the SYBR Green Pro Taq HS Premixed qPCR Kit (AG11733, Accurate Biology). GAPDH served as the internal control. Relative gene mRNA expression was determined applying the 2^−ΔΔCt^ method with primers (*Homo sapiens* and *Mus musculus*) detailed in Table S1.

### 2.11 Flow cytometry assay

Cell cycle distribution and apoptosis were analysed by flow cytometry analysis using an Annexin V‐FITC/PI Apoptosis Detection Kit (A211, Vazyme, China). For cell cycle analysis, cells were washed with precooled PBS, fixed by 70% ethanol, treated with RNaseA, and stained with propidium iodide (PI) prior to analysis. For apoptosis detection, collected cells (approximately 1 × 10⁶ cells each sample) were washed, resuspended in 100 µL binding buffer, stained with 5 µL of Annexin V‐FITC and 5 µL of PI for 10 min under dark ambient conditions, then supplemented with 400 µL binding buffer before analysis. Samples underwent immediate FACScan flow cytometry (BD Biosciences) with subsequent FlowJo analysis (v10.8.1; BD).

### 2.12 Dual luciferase reporter assays

Luciferase activity was quantified with the Dual‐Luciferase Reporter Assay System Kit (E1960, Promega, USA) according to the manufacturer's instructions. The cells received co‐transfection with both the luciferase reporter vector and a Renilla luciferase control plasmid. Firefly luciferase luminescence values were normalised to Renilla luciferase readings for each transfected sample. Three independent biological replicates were performed.

### 2.13 Chromatin immunoprecipitation (ChIP) assays

ChIP assay was standardised with the BeyoChIP™ ChIP Assay Kit (P2080S, Beyotime, China) according to the manufacturer's protocol. c‐Myc antibodies were obtained from HUABIO (HA721182, China). The primer sequences used for ChIP analysis are detailed in Table S4. Immunoprecipitated DNA was quantified by RT‐qPCR, with results expressed as a percentage of input DNA. Triplicate biological replicates characterised each experiment.

### 2.14 Animal models

All animal procedures were reviewed and authorised by the Institutional Animal Care and Use Committee of Nanjing Medical University (Approval Number: IACUC‐2409020), and were carried out in strict compliance with the guidelines outlined in the Guide for the Care and Use of Laboratory Animals. Four‐week‐old female C57BL/6 immunocompetent mice were purchased from GemPharmatech Co., Ltd. and received orthotopic injections of Py8119 cells at a concentration of 1×10⁶ cells per mouse. Treatments began one week post‐injection. Mice were assigned into four separate groups randomly: shControl, shControl receiving PD‐L1 inhibitor (10 mg/kg, twice weekly), shc‐Myc receiving PD‐L1 inhibitor, and shc‐Myc receiving both Salirasib (10 mg/kg, every other day) and PD‐L1 inhibitor. At the end of the experiment, mice were euthanised, and tumours were excised and weighed. Additionally, lung metastasis models were established by intravenously injecting Py8119 cells (1 × 10⁶ cells) into separate female C57BL/6 immunocompetent mice (aged 6 weeks). Tumour metastasis was regularly monitored using the IVIS® Lumina II imaging system (Caliper Life Sciences, MA, USA).

### 2.15 Immunohistochemical (IHC) staining and haematoxylin and eosin (H&E)

Tumour and primary organ tissue sections, fixed in formalin and paraffin‐embedded, underwent H&E staining for morphological assessment and were imaged using light microscopy. FFPE tumour sections were deparaffinised, rehydrated, and underwent citrate‐based antigen retrieval for IHC. Following endogenous peroxidase blockade (3% H_2_O_2_), sections received overnight incubation (4°C) with primary antibodies against Ki‐67 (1:200, ab16667, Abcam) and MCM2 (1:800, #3619, CST), HRP‐conjugated secondary antibodies (Servicebio), and DAB visualisation. To identify apoptotic cells, we conducted TUNEL staining assay using a DAB TUNEL Apoptosis Detection Kit (Servicebio, China). Post‐permeabilisation, sections were incubated with agent mixture (37°C, 1 h), counterstained with DAPI, and imaged by fluorescence microscopy.

### 2.16 Immunofluorescence (IF) staining

Murine breast tumour paraffin sections underwent immunofluorescence staining with antibodies against CD11b, CD80, and CD206 (all from Abcam). Sections were incubated with secondary antibodies (37°C, 1 h), counterstained with DAPI (G1012, Servicebio, China), and imaged by confocal microscopy (LSM700, ZEISS, Germany).

### 2.17 Bioinformatic analysis

Breast invasive carcinoma (BRCA) datasets were obtained from The Cancer Genome Atlas (TCGA; https://cancergenome.nih.gov/), and TNBC cases were identified by negative ER, PR, and HER2 expression. Gene expression profiles were analysed, and correlations between MYC and other genes were assessed. Patients were stratified by gene expression levels for Kaplan–Meier survival and Kyoto Encyclopedia of Genes and Genomes (KEGG) pathway enrichment analysis. Besides, groups stratified by high/low expression were delineated based on median gene expression values, ensuring both optimal group separation and balanced sample sizes for robust statistical analysis. RNA sequencing (RNA‐seq) data from TNBC cells were also analysed to identify differentially expressed genes (DEGs), with KEGG enrichment performed separately for upregulated and downregulated DEGs.

### 2.18 Statistical analysis

We conducted Statistical analyses using GraphPad Prism 10.1.2 (San Diego, CA, USA). Data represent mean ± SD. Two‐group comparisons used Student's *t*‐test; multigroup comparisons employed one‐way ANOVA. Significance threshold was *p* < .05 (two‐sided). Each experiment included three biological replicates.

## 3 RESULT

### 3.1 Ras inhibitor Salirasib induces synthetic lethality in TNBC cell lines upon c‐Myc knockdown

**FIGURE 1:**
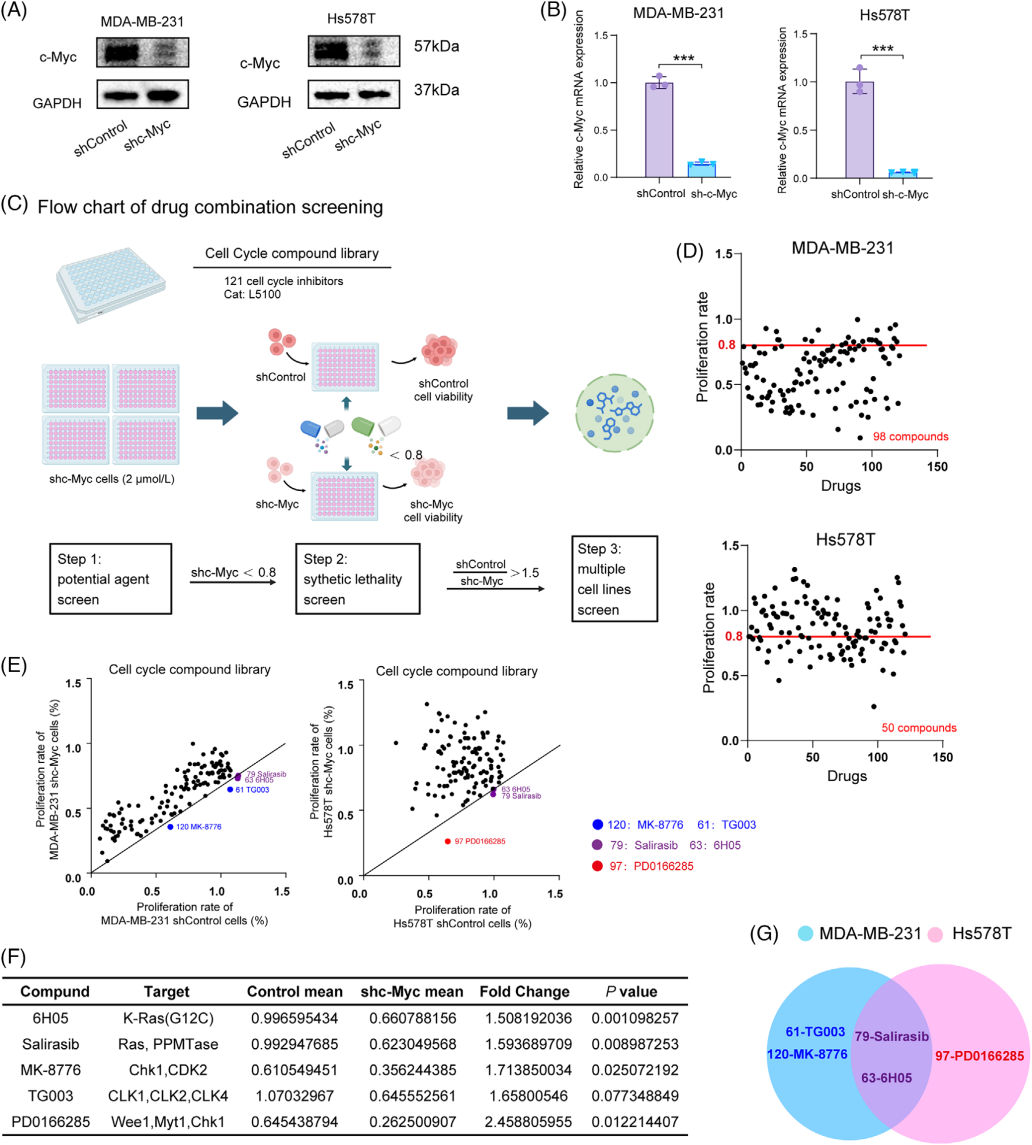
Combinatorial screen reveals c‐Myc–targeted synergists for TNBC therapy. (A, B) Western blot and RT‐PCR confirmed c‐Myc knockdown in MDA‐MB‐231 and Hs578T cells, respectively. (C) Identifying c‐Myc‐targeted compounds enhancing TNBC therapy via drug screening. (D) Dot plot of 98 and 50 inhibitory candidates respective among screening candidates. Data points represent individual compounds. (E) The dot plot displays the number of inhibitors that have been found to have potent synergistic effects with targeted inhibition of c‐Myc. The blue dots represent the drugs that have synergistic effects only in MDA‐MB‐231 shc‐Myc cells, the red dots show the drugs that have synergistic effects only in Hs578T shc‐Myc cells, and the purple dots display the drugs that have synergistic effects in both TNBC cells. (F) List of the total candidates after the synthetic lethality screen in two TNBC cell lines. (G) Two overlapping candidates were identified in the two TNBC cells after the synthetic lethality screen. Data were summarised as means  ±  SD in (B). *p  <  .05; **p  < .01; ***p  <  .001.

c‐Myc expression was screened in four TNBC lines. Human MDA‐MB‐231 and Hs578T were selected for in vitro studies, and murine Py8119 for in vivo validation, based on protein expression profiles (Figure S1A). To identify drugs that induce synthetic lethality upon c‐Myc knockdown, we transduced these TNBC cell lines with shRNAs targeting c‐Myc (Figures 1A and B and S1B and C). We adopted a strategy for screening drug combinations to evaluate the impact of synergies.^20^ Previous research has indicated that aggressive TNBC with elevated c‐Myc expression exhibits unique sensitivity to CDK inhibition.^13^, ^15^, ^21^ Therefore, we chose a cell cycle compound drug library consisting of 121 compounds to explore the underlying mechanisms. The screening process was designed to identify potential small molecule inhibitors that trigger synthetic lethality in the context of c‐Myc knockdown. We structured the experiment into three main steps: initial screening of candidate agents, identification of synthetic lethal interactions, and subsequent validation across various cell lines (Figure 1C). In the initial phase, we evaluated the inhibitory potential of each compound against the proliferation of c‐Myc knockdown TNBC cells. Specifically, 98 and 50 agents, respectively, demonstrated at least 20% inhibitory activity against the two selected c‐Myc knockdown cell lines (Figure 1D). In the second step, agents that resulted in a cell viability ratio (shControl/shc‐Myc) exceeding 1.5 were considered to exhibit synergistic effects with c‐Myc targeting and were thus selected for further investigation (Figure 1E). In the final step, four compounds in MDA‐MB‐231 cells and three compounds in Hs578T exhibited significant synergistic efficacy with inhibiting c‐Myc. Consequently, we identified five compounds exerting synergistic efficacy upon c‐Myc suppression (Figure 1F). Notably, two overlapping compounds – Salirasib and 6H05, both Ras inhibitors – demonstrated consistent synergistic effects in both TNBC cells, highlighting their potential as robust candidates for further investigation (Figure 1G).

### 3.2 Salirasib synergistically suppressed TNBC cell proliferation upon c‐Myc inhibition

**FIGURE 2:**
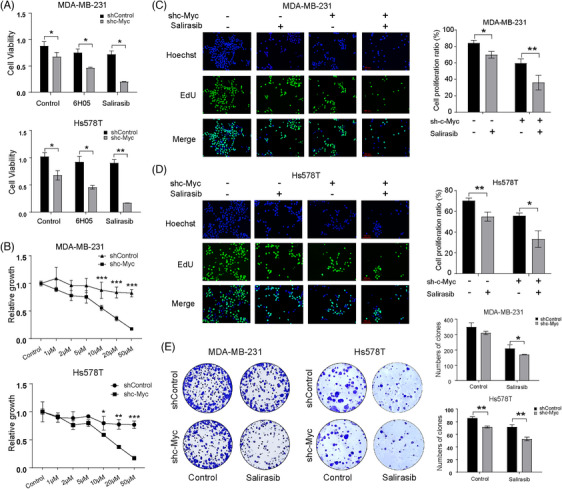
Characterisation of the anti‐TNBC impact of the combined use of Salirasib with targeted inhibition of c‐Myc. (A) Cell viability of two TNBC cells treated with Salirasib and 6H05 respectively was detected via CCK‐8. Salirasib exhibited a more pronounced effect compared to 6H05. (B) CCK8 assay elucidated the alterations in viability of TNBC cells exposed to different concentrations of Salirasib. The concentration of Salirasib corresponding to 50% cell viability in TNBC shc‐Myc cells was approximately 10 µM. (C, D) EdU cell proliferation assay was used to evaluate the viability of TNBC cells following treatment with Salirasib in combination with targeting c‐Myc. (E) Colony formation assay was applied to exam long‐term TNBC cell survival after after the treatment of Salirasib combined with targeting c‐Myc. Data were summarised as means  ±  SD. *p  <  .05; **p  <  .01; ***p  <  .001.

Cell proliferation assay with CCK‐8 was employed to verify the synergistic effect of c‐Myc knockdown when combined with two distinct agents: Salirasib and 6H05. Ras, a crucial small GTPase belonging to the Ras superfamily and operating via dynamic GTP/GDP cycling, holds a pivotal regulatory function in the cell cycle progression of TNBC.^22^ Numerous studies have elucidated the complementary biological roles of Ras and c‐Myc, particularly their coordinated induction and stabilisation of cell cycle proteins.^23^, ^24^ Notably, Salirasib and 6H05, two Ras inhibitors, exhibit different mechanisms: Salirasib disrupts Ras signalling to hinder tumour growth, whereas 6H05 selectively targets mutant K‐Ras (G12C) through allosteric inhibition. Following this, we assessed the viability of TNBC cells exposed to Salirasib and 6H05, respectively, to compare the proliferation rates between shControl and shc‐Myc cells. Both compounds demonstrated comparable inhibitory effects on shControl cells. However, Salirasib showed a significantly enhanced suppressive effect on shc‐Myc cell proliferation in TNBC cells (Figure 2A). Consequently, Salirasib was chosen for further investigation due to its remarkable synergy with c‐Myc knockdown. Interestingly, 6H05 is a mutation‐selective inhibitor that targets the KRAS(G12C) switch‐II pocket by reacting with the mutant cysteine residue.^25^ However, the two TNBC cell lines engaged lack KRAS(G12C) mutations: MDA‐MB‐231 carries KRAS(G13D) and Hs578T harbours HRAS(G12D) mutation.^26^ The observed suppression with 6H05 therefore likely reflects dose‐dependent off‐target effects rather than on‐target activity, consistent with the concept of concentration‐dependent ‘selectivity windows’ for KRAS(G12C) inhibitors.^27^, ^28^, ^29^ That is, as the concentration increases into the upper micromolar range, even highly selective compounds can exhibit non‐specific effects in cell viability assays. Although Salirasib exhibited some inhibitory effect on the control group, shc‐Myc cells proved to be more sensitive to Salirasib at a concentration of 10 µM when compared to shControl cells under the same treatment conditions (Figure 2B). This finding was further corroborated through EdU cell proliferation assays and colony formation assays (Figure 2D and E). These findings collectively demonstrate that the combination of Salirasib and c‐Myc knockdown produces a synergistic antiproliferative effect in TNBC cells, significantly exceeding the efficacy of either agent alone.

### 3.3 Targeting c‐Myc combined with Salirasib synergistically induced G1/S phase arrest and apoptosis in TNBC cells

**FIGURE 3:**
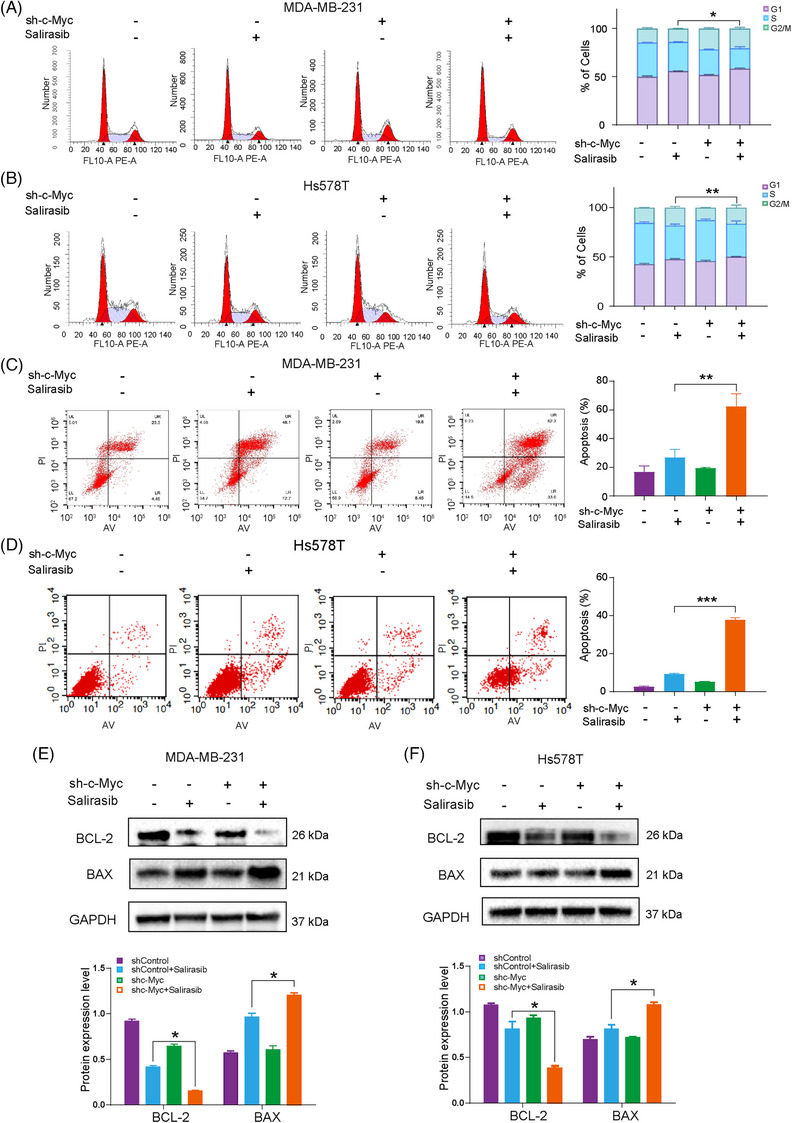
Combination of Salirasib with targeting c‐Myc synergistically induces cycle arrest and apoptosis. (A, B) Flow cytometry analysis of the percentage of the cell cycle of MDA‐MB‐231 and Hs578T cells after pretreatment with Salirasib for 48 h. (C, D) Flow cytometry analysis of the proportion of apoptotic MDA‐MB‐231 and Hs578T cells treated with Salirasib for 48 h. (E, F) Apoptosis protein (BCL‐2 and BAX) expression in TNBC cells after 48‐h Salirasib treatment. Data were summarised as means  ±  SD. *p  <  .05; **p  <  .01; ***p  <  .001.

Ras proteins, which are small GTPases, hold a pivotal position in cellular signalling cascades, influencing cell growth, proliferation, and survival.^30^ Based on this understanding, we hypothesised that the synergistic effect of two treatments could potentially modulate the cell cycle. Through flow cytometry analysis, a pronounced increase was observed in cells arrested at the G1/S phase when treated with a combination of c‐Myc knockdown and Salirasib, as compared to either treatment alone (Figure 3A and B). This cell cycle arrest might consequently lead to significant cell apoptosis. Our findings indicate that the combined approach of targeting c‐Myc inhibition and Salirasib administration exacerbates cell apoptosis more than individual treatments (Figure 3C and D). Furthermore, elevated expression of BAX and a concomitant reduction in BCL‐2 were observed in the combination treatment group, suggesting an augmentation of cancer cell apoptosis through this combinatorial approach (Figure 3E and F). The results suggest that the synergy of targeting c‐Myc with Ras inhibitor induced cell cycle arrest while simultaneously triggering apoptosis, thereby elucidating the mechanistic foundation of their synergistic antitumour activity.

### 3.4 Targeting c‐Myc combined with Salirasib synergistically suppressed TNBC cell invasion and migration

**FIGURE 4:**
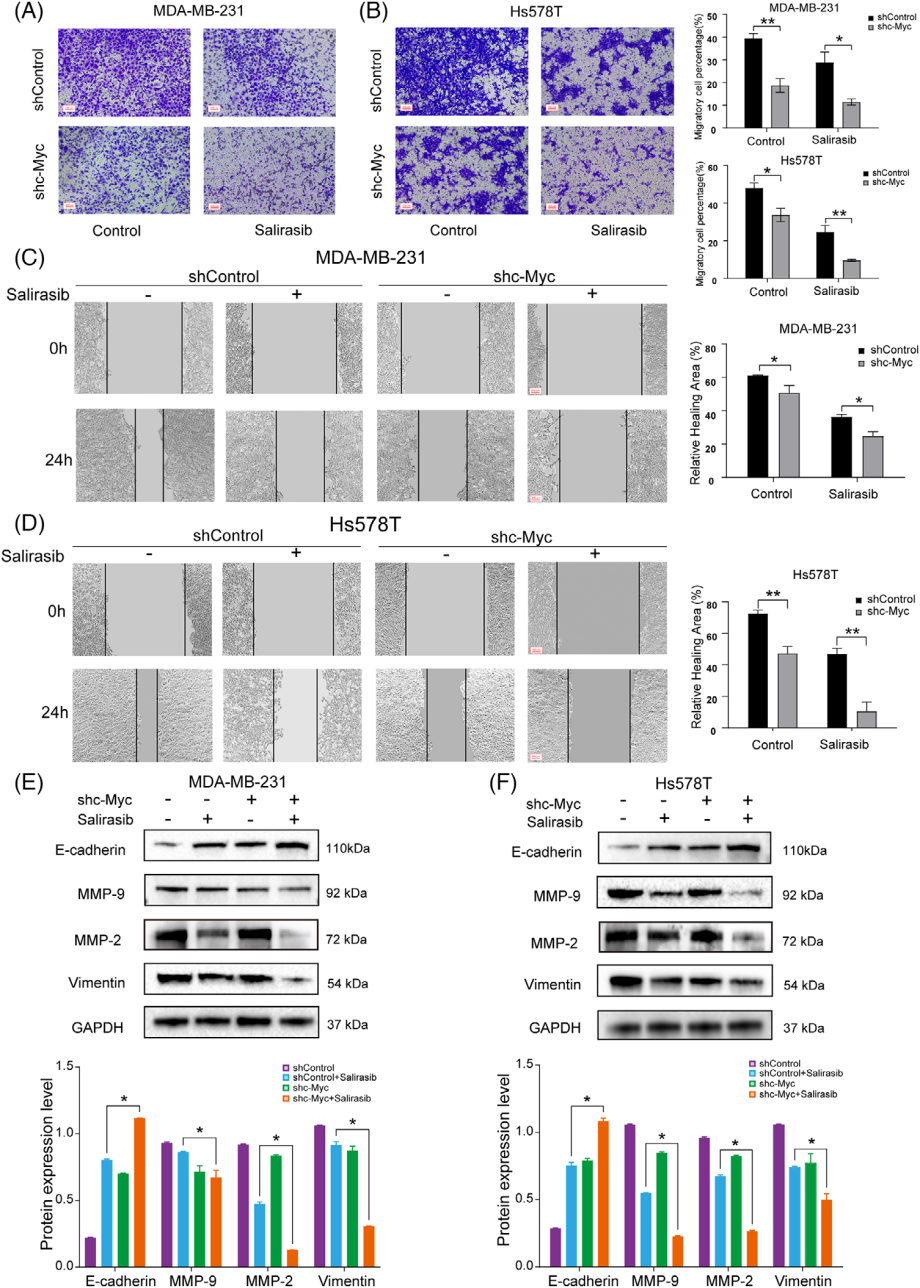
Combination of Salirasib with targeting c‐Myc synergistically inhibited TNBC cell migration and invasion. (A, B) Transwell assays were utilised to examine cell invasion of MDA‐MB‐231 and Hs578T cells after the treatment of Salirasib with targeting c‐Myc. (C, D) The scratch wound assays were applied to examine the cell migration in indicated groups of MDA‐MB‐231 and Hs578T cells. (E, F) Migration‐related protein (E‐cadherin, MMP‐9, MMP‐2, and Vimentin) expression in TNBC cells exposed to Salirasib after 48‐h Salirasib treatment. Data were summarised as means  ±  SD. *p  <  .05; **p  <  .01.

TNBC's aggressive and metastatic nature underscores the critical therapeutic significance of repressing tumour cell invasion and distant metastasis. To elucidate this, transwell assay was employed, revealing that the combination therapy cohort suppressed cell invasion significantly as opposed to mono‐therapy cohorts (Figure 4A and B). Additionally, a marked reduction was observed in the migratory capacity of TNBC cells within the synergistic treatment group via wound healing assay (Figure 4C and D). Further analysis focused on migration proteins expression, specifically E‐cadherin, MMP‐9, MMP‐2, and Vimentin. Both the targeted inhibition of c‐Myc and Salirasib monotherapy enhanced E‐cadherin expression while suppressing MMP‐9, MMP‐2, and Vimentin expression. Notably, the combined therapy exhibited more pronounced effects on the expression levels of these proteins (Figure 4E and F). These findings suggest that the synergy of targeting c‐Myc and Salirasib may effectively suppress TNBC cell invasion and migration. Moreover, our results indicate a significant reduction in EMT in shc‐Myc TNBC cells exposed to Salirasib. This reduction was markedly more pronounced than either monotherapy, further confirming the synergistic effect between these two treatments. In summary, these findings highlight that combined c‐Myc inhibition and Ras pathway targeting not only suppress TNBC cell invasion and migration, but also present a promising strategy to limit TNBC metastasis.

### 3.5 MCM2 is a potential mediator of synthetic lethality induced by co‐targeting c‐Myc and Ras signalling

Based on the TCGA database, we identified 119 TNBC patients among 1059 primary breast cancer cases and assessed the prognostic significance of MYC, KRAS, and CD274 (PD‐L1) mRNA expression. Kaplan–Meier analysis demonstrated that elevated MYC and KRAS expression was independently related poorer overall survival (Figure S2A and B). It is worth noting that concurrent high expression of both MYC and KRAS was associated with even worse survival outcomes (Figure S2C). Although low PD‐L1 expression was generally linked to poorer survival compared to high PD‐L1 expression (Figure S2D), the simultaneous low expression of MYC, KRAS, and PD‐L1 conferred a particularly pronounced survival advantage in comparison to patients with high expression of all three markers (Figure S2E). Additionally, we extended our analysis to explore the effect of MYC on KRAS and PD‐L1 expression, observing positive correlations of MYC mRNA levels with of KRAS and PD‐L1 expression in TNBC patients (Figure S3A and B). Following this, we computationally simulated the effects of combining c‐Myc targeting with the Ras inhibitor Salirasib by stratifying TNBC patients based on MYC and KRAS expression. The volcano plot highlighted significant differences between the dual‐low MYC/KRAS and dual‐high MYC/KRAS groups, revealing 2167 DEGs, comprising 1664 upregulated and 503 downregulated genes (Figure S3C). We performed functional enrichment analysis to delve deeper into the significant pathways associated with these DEGs. KEGG pathway analysis of the upregulated DEGs uncovered enrichment in Neutrophil extracellular trap formation and cytokine‐cytokine receptor interaction pathways (Figure S3D). This pattern intriguingly implied that the dual‐low expression of MYC and KRAS in TNBC might potentiate immune activation. On the other hand, the analysis of downregulated DEGs predominantly revealed enrichment in mitogen‐activated protein kinase (MAPK) signalling pathways and the regulation of cell cycle (Figure S3E). Low KRAS expression could underlie the MAPK signalling disruption, while low MYC expression might contribute to cell cycle dysregulation via CDK activity inhibition. Recent studies indicate that MYC and KRAS jointly contribute to immunosuppression in TNBC and targeting both c‐Myc and Ras offers potential for enhancing immunotherapy effectiveness by modulating oncogenic and immune networks.^24^, ^31^, ^32^ To further explore this, we assessed the immune infiltration landscape of the TME across different levels of MYC/KRAS expression. The correlation matrix revealed weak to moderate correlations between various tumour‐infiltrating immune cell (TIIC) subsets, underscoring the intricate crosstalk mediated by c‐Myc and Ras‐associated mechanisms in TNBC (Figure S3F). While chemotherapy or radiotherapy may affect patient survival, this analysis could not incorporate these clinical variables, presenting a limitation. For all this, the TCGA datasets analysis still provided a novel perspective and solid support for our study.

**FIGURE 5:**
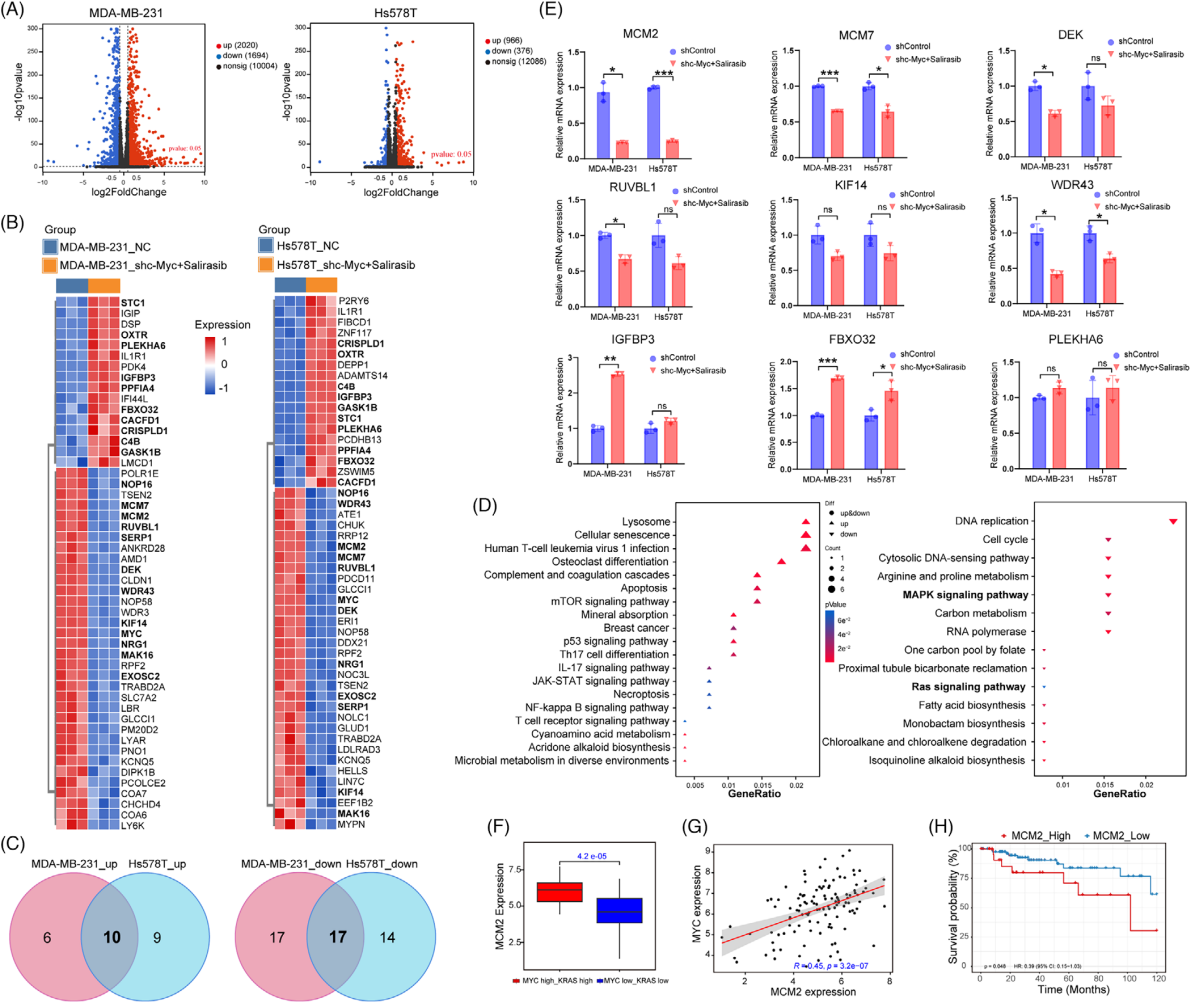
MCM2 expression was related to anti‐TNBC effects by targeted inhibition of c‐Myc combined with Salirasib. (A) Volcano plot of DEGs in two TNBC cells comparing the treatment group (c‐Myc inhibition + Salirasib) and the control group. (B) Heatmap analysis of the TOP 50 DEGs in both TNBC cell lines. (C) Among the TOP 50 DEGs, the Venn diagram indicated that both cell lines shared 10 genes with increased expression and 17 with decreased expression. (D) KEGG enrichment analysis revealed that upregulated DEGs enriched in apoptosis and immune related pathways, while downregulated DEGs were linked to DNA replication, cell cycle, MAPK, and Ras signalling pathways. (E) mRNA expression levels of cell cycle‐related genes derived from the 10 upregulated and 17 downregulated DEGs. (F) MCM2 expression in TNBC patients stratified by MYC/KRAS expression levels (TCGA dataset). (G) Relationship between MCM2 and MYC expression in TNBC from the TCGA database. (H) Correlation between MCM2 mRNA expression level and overall survival in TNBC patients from the TCGA database. Data were summarised as means  ±  SD in (E). *p  <  .05; **p  <  .01; ***p  <  .001.

To comprehensively profile the transcriptomic landscape of TNBC, we conducted RNA‐seq on two TNBC cell lines after stably knocking down c‐Myc and treating them with Salirasib for 48 h. Using the RNA‐seq data, we performed a differential expression analysis to pinpoint cell cycle‐related DEGs between the control and treatment groups. Volcano plots revealed 3714 DEGs (2020 upregulated and 1694 downregulated) in MDA‐MB‐231 and 1342 DEGs (966 increased and 376 decreased) in Hs578T (Figure 5A). Top 50 genes from each cell line's DEGs were visualised through heatmaps, and among these, we identified 10 commonly upregulated and 17 commonly downregulated DEGs using Venn diagrams (Figure 5B and C). Additionally, KEGG enrichment analysis of these common DEGs showed that the genes with increased expression were enriched in apoptosis and immune‐related pathways, whereas the genes with decreased expression exhibited enrichment in cell cycle, DNA replication, MAPK, and Ras signalling pathways (Figure 5D). Based on these findings, we prioritised cell cycle‐associated genes, including MCM2, MCM7, DEK, RUVBL1, KIF14, WDR43, IGFBP3, FBXO32, and PLEKHA6, for further mRNA validation using RT‐PCR. Consistent with our transcriptomic analyses, qPCR validation further confirmed that both MCM2 and MCM7 were downregulated notably following combined treatment in both cell lines (Figure 5E). Importantly, the reduction in MCM2 expression was more pronounced and statistically significant, whereas MCM7 also exhibited a significant decrease but to a lesser extent. Given that MCM2 and MCM7 are integral subunits of the MCM2–7 helicase complex, their concurrent suppression suggests a cooperative impairment of DNA replication licensing. Nevertheless, because MCM2 showed the most robust downregulation and has been strongly associated with c‐Myc activity and TNBC prognosis (Figure 5G and H), we selected MCM2 as the primary representative target in our mechanistic exploration. MCM2, a pivotal subunit of the MCM complex, is fundamental for the initiation of DNA replication.^33^ Elevated expression of MCM2 in breast cancer patients is tightly linked to a poor prognosis.^34^ In this study, we delved deeper into TNBC datasets from TCGA to understand the expression patterns of MCM2. As expected, MCM2 showed significantly higher expression levels in the dual‐high MYC/KRAS subgroup (Figure 5F), positively correlating with MYC expression (Figure 5G). Kaplan–Meier survival analysis further revealed that lower MCM2 expression was associated with better overall survival in patients with TNBC (Figure 5H). Taken together, our results strongly suggest a close association between MCM2 expression, MYC activity, and prognostic outcomes in TNBC.

**FIGURE 6:**
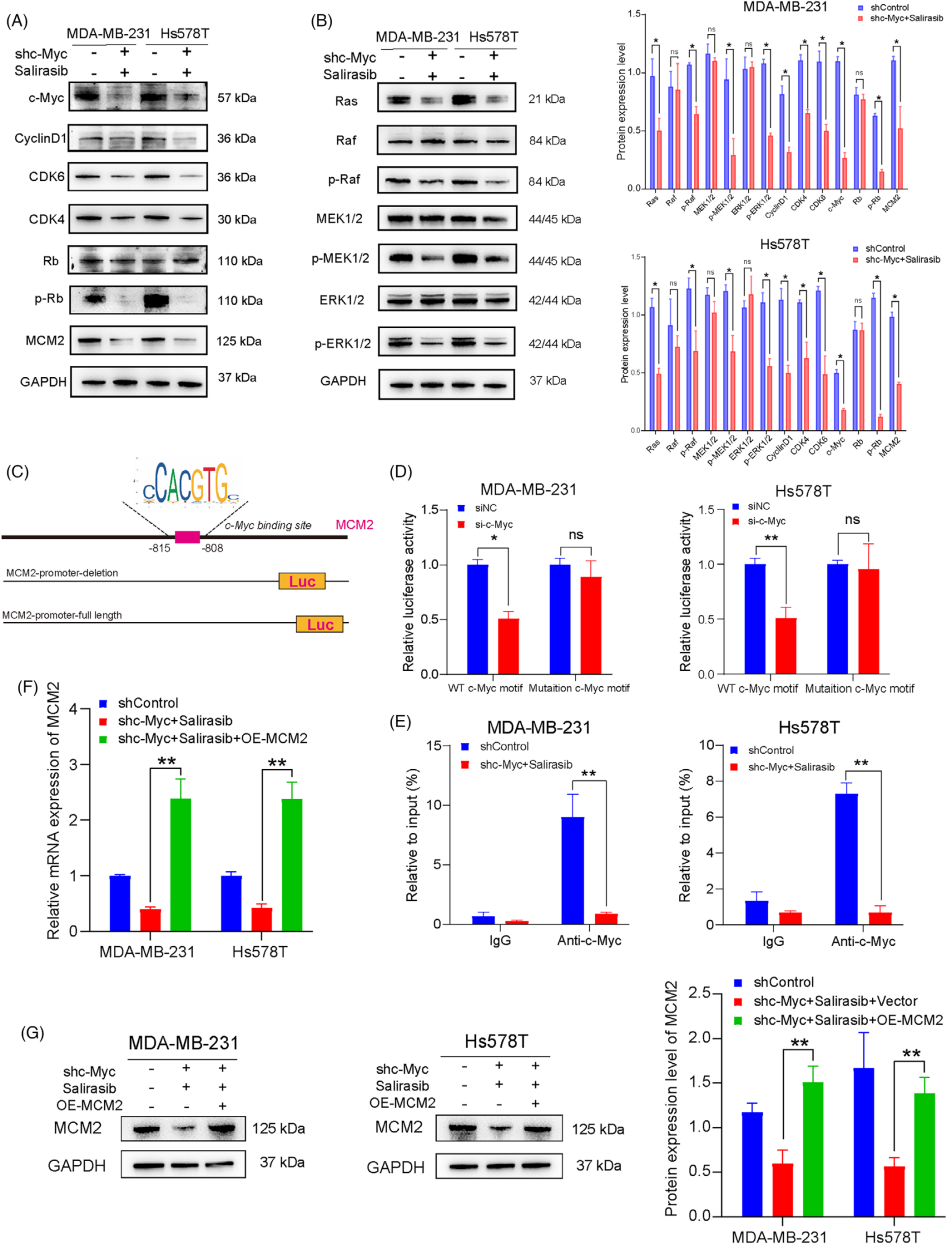
MCM2 mediated as the key effector of synthetic lethality by dual targeting of c‐Myc and the Ras pathway. (A) Alterations in expression of c‐Myc downstream proteins after combined treatment of targeting c‐Myc and Salirasib. (B) Alterations in expression of Ras downstream proteins after combined treatment of targeting c‐Myc and Salirasib. (C) Schematic illustration of c‐Myc binding sites within the MCM2 promoter region. (D) Dual‐luciferase assay was employed to detect MCM2 promoter activity. (E) ChIP assay was performed to evaluate c‐Myc recruitment to the MCM2 promoter. (F, G) RT‐PCR and Western blot confirmed MCM2 expression level for rescue experiments. Data were summarised as means  ±  SD. *p  < .05, **p  < .01.

The aforementioned results indicated that MCM2 functions as a pivotal terminal regulator and effector in the Ras‐MAPK signalling pathway. Simultaneously, it is modulated by c‐Myc, jointly regulating the cell cycle shift from the G1 to the S phase. Our study emphasises the synergistic interplay between c‐Myc targeted inhibition by knockdown and Ras signalling blockade through Salirasib. Precisely, the suppression of Ras signalling by Salirasib augmented the inhibitory impact on c‐Myc, collaboratively diminishing MCM2 expression. This ultimately led to a notable G1‐phase arrest and hindered progression to the S‐phase. These outcomes underscore the promising therapeutic approach of concurrently targeting c‐Myc and Ras signalling pathways to attain heightened anti‐proliferative efficacy. Based on these findings, we aimed to further investigate the synergistic mechanisms that underlie the combined targeting of c‐Myc and Salirasib‐induced Ras inhibition. Salirasib exerts its antitumour effects by interfering with the localisation of active Ras proteins to the plasma membrane, thereby attenuating downstream Ras signalling cascades, specifically the PI3K/Akt/mTOR pathways and Raf/MEK/ERK, essential for tumour cell proliferation and long‐term survival. In light of these observations, we sought to explore the combined impact of Salirasib‐induced Ras inhibition and targeted suppression of c‐Myc in TNBC cells. By specifically disrupting the interaction between active Ras proteins and the plasma membrane, Salirasib demonstrates its antitumour potency, inhibiting downstream Ras‐dependent signalling cascades. Incorporating our previous bioinformatics analysis, we hypothesised that the combined targeting of Ras via Salirasib and c‐Myc inhibition could effectively downregulate MCM2 expression. This, in turn, could lead to compromised proliferation, cell cycle arrest at the G1/S transition, and enhanced apoptosis in TNBC cells. This initial hypothesis was further substantiated through subsequent western blot experiments conducted on two TNBC cell lines (Figure 6A and B). Since c‐Myc functions as a critical transcription factor, we examined the MCM2 promoter for potential binding sites to decipher the regulatory mechanisms governing transcription. A candidate binding locus for c‐Myc site was identified within the core promoter region upstream of the MCM2 gene (Figure 6C), implying that MCM2 may be directly activated by c‐Myc. Luciferase reporter assays further demonstrated that c‐Myc knockdown selectively inhibited the wild‐type promoter while the c‐Myc‐mutated promoter remained unaffected in both TNBC cells (Figure 6D). Subsequent ChIP assays verified c‐Myc's direct binding to the MCM2 promoter, with reduced binding abundance observed upon c‐Myc inhibition in the two TNBC cells (Figure 6E). It should be noted that the purpose of these experiments was to establish whether c‐Myc can directly regulate MCM2 transcription, rather than to comprehensively identify all potential binding motifs within the MCM2 promoter. While additional low‐scoring binding motifs may exist, the current study focused on establishing the presence of direct transcriptional regulation, not on exhaustively mapping all binding sites.

**FIGURE 7:**
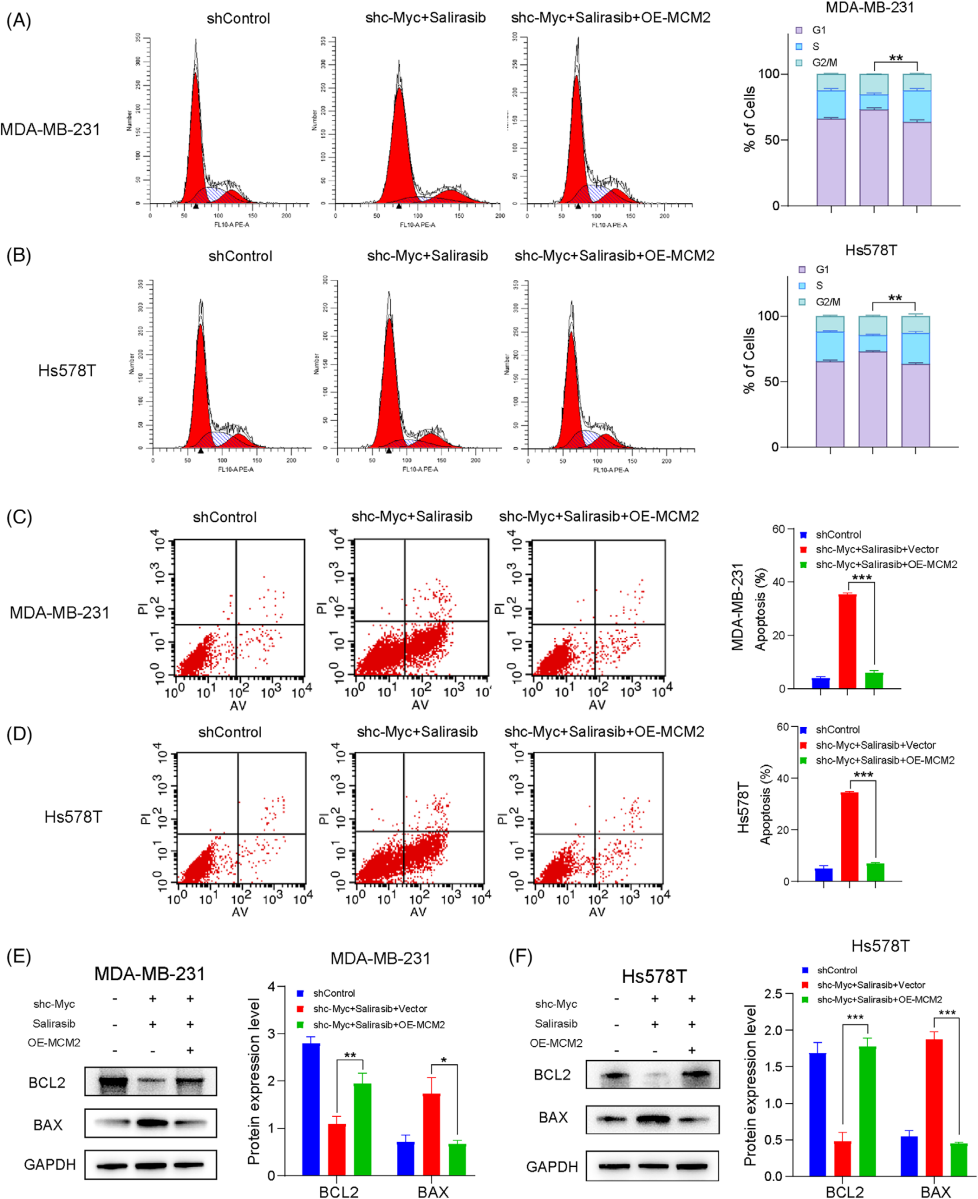
Restoration of MCM2 reverses cell cycle arrest and apoptosis induced by the synergism of Salirasib with targeting c‐Myc. (A, B) Flow cytometry analysis of the percentage of the cell cycle of MDA‐MB‐231 and Hs578T cells with different pretreatments. (C, D) Flow cytometry analysis of the proportion of apoptotic TNBC cells exposed to different pretreatments. (E, F) Apoptosis protein (BCL‐2 and BAX) expression of TNBC cells exposed to different pretreatments. Data were summarised as means  ±  SD. *p  < .05; **p  < .01; ***p  < .001.

Then, we further confirmed the expression of MCM2 for subsequent rescue experiments (Figure 6F and G). And the cell viability and proliferation inhibited by the synergy of Salirasib and targeting c‐Myc w ere restored after MCM2 overexpression rescue (Figure S4A–C), indicating that MCM2 may be a key downregulating factor under the dual inhibition of c‐Myc and Ras. Besides, cells arrested at G1/S phase and apoptosis were observed diminished significantly once MCM2 overexpression rescue (Figure 7A–D). And the observed changes were also accompanied by corresponding alterations in apoptosis‐related protein expression. The rescue group exhibited decreased BAX expression coupled with increased BCL‐2 expression (Figure 7E and F), denoting reduced TNBC cell apoptosis after restoring MCM2 expression. Hence, the above functional validation assays established MCM2 as a critical regulator of G1/S phase arrest and apoptotic induction.

### 3.6 Targeting c‐Myc synergises with Salirasib to enhance immunotherapy efficacy in vivo

**FIGURE 8:**
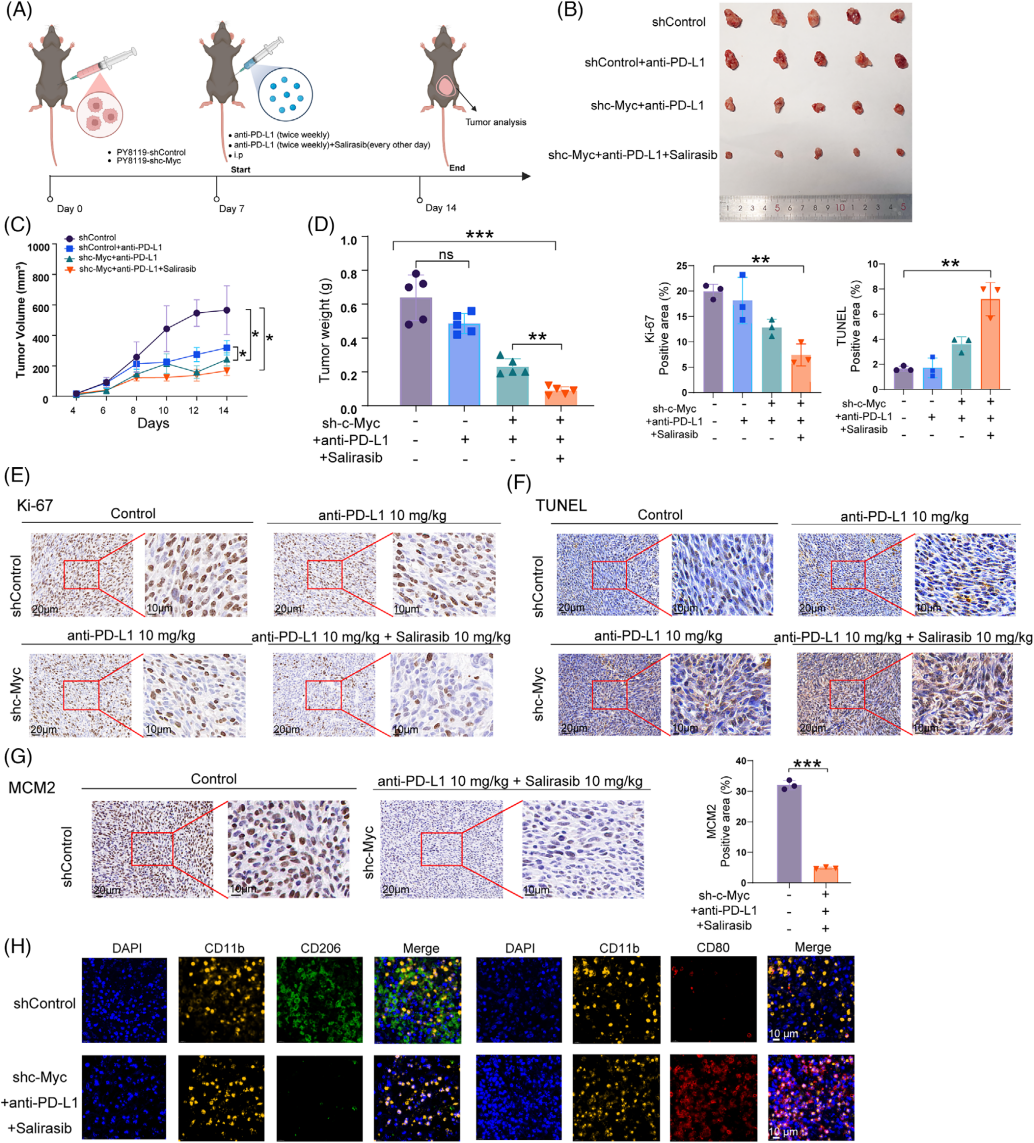
Targeting c‐Myc synergises with Salirasib significantly enhanced the anti‐TNBC effect of immunotherapy in murine orthotopic TNBC model. (A) Timeline of establishment and treatment of murine models of primary TNBC. (B) Tumour size, (C) tumour volume and (D) tumour weight measured for each treatment group. (E, F) Immunohistochemical staining of Ki‐67 and TUNEL showed that targeting c‐Myc combined with Salirasib significantly potentiated immunotherapy‐mediated tumour control in vivo. (G) Immunohistochemical staining revealed a reduced expression of MCM2 in the shc‐Myc group treated with the PD‐L1 inhibitor and Salirasib compared to the shControl group. (H) Immunofluorescence staining revealed reversed polarisation of macrophages mediated by the synergy of Salirasib combined with targeting c‐Myc. Data were summarised as means  ±  SD. *p  < .05; **p  < .01; ***p  < .001.

To evaluate the therapeutic potential of targeting c‐Myc with Salirasib in TNBC, we developed an orthotopic murine TNBC model (Figure 8A). Mice underwent randomisation into four therapeutic cohorts: shControl, shControl with PD‐L1 inhibitor, shc‐Myc with PD‐L1 inhibitor, and shc‐Myc with both Salirasib and PD‐L1 inhibitor. PD‐L1 inhibitor administrations occurred twice weekly, whereas Salirasib was given every other day. The shControl group received an equivalent volume of saline as placebo. Quantitative analysis of tumour weight and size revealed that the shc‐Myc group treated with both Salirasib and PD‐L1 inhibitor achieved superior tumour suppression efficacy compared to alternative regimens. This finding highlights the robust antitumour immunity achieved through the synergy of c‐Myc suppression and Salirasib. Although the PD‐L1 inhibitor exhibited limited efficacy when used alone, its combination with Salirasib significantly augmented the therapeutic outcome, surpassing the effects of either treatment alone (Figure 8B–D). IHC results further supported these findings, showing the greatest reduction in the proliferation indicator Ki‐67 and the most substantial increase in the apoptosis indicator TUNEL in the shc‐Myc group receiving dual treatment with the PD‐L1 inhibitor and Salirasib (Figure 8E and F).

We conducted a further investigation into the expression of MCM2 in the shc‐Myc group that underwent PD‐L1 inhibitor and Salirasib treatment, as well as in the control group. Our findings revealed a notable decrease in MCM2 expression in the shc‐Myc group that received the combined treatment compared to the control group. This observation aligns with the RNA‐seq analysis conducted on TNBC cell lines (Figure 8G). Since the treatment regimen encompassed ICIs and demonstrated significant therapeutic efficacy, we sought to explore the changes in TME using a TNBC orthotopic tumour model. To resolve macrophage repolarisation dynamics in TME, multiplex immunofluorescence (mIF) was conducted on FFPE murine mammary carcinoma sections. These tumour samples were precisely sliced and subsequently stained using DAPI, as well as antibodies against CD11b, CD206, and CD80. Specifically, we detected an increase of CD11b^+^CD80^+^ (M1‐like) macrophages together with a decrease of CD11b^+^CD206^+^ (M2‐like) macrophages in the tumour tissue of the shc‐Myc group treated with PD‐L1 inhibitor and Salirasib compared with the control group, resulting in an elevated CD80/CD206 ratio (Figure 8H). This remodelling of tumour immune microenvironment (TIME) with focus on tumour associated macrophages (TAMs) polarisation aligned with the above RNA‐seq results and TCGA dataset analyses. Based on KEGG enrichment results from both transcriptomic and TCGA‐based analyses, we drew upon immune‐related pathways enriched among the upregulated DEGs – including the T cell receptor signalling pathway (TCR), JAK–STAT signalling, NF‐κB signalling, lysosome activity, cytokine–receptor interactions, and neutrophil extracellular traps – to interpret macrophage plasticity and the observed improvement in ICI responsiveness. These results provide compelling evidence that Salirasib exhibits a synergistic interaction with c‐Myc‐targeted therapy, which potently facilitates the repolarisation of TAMs toward the antitumoural M1 phenotype.

**FIGURE 9:**
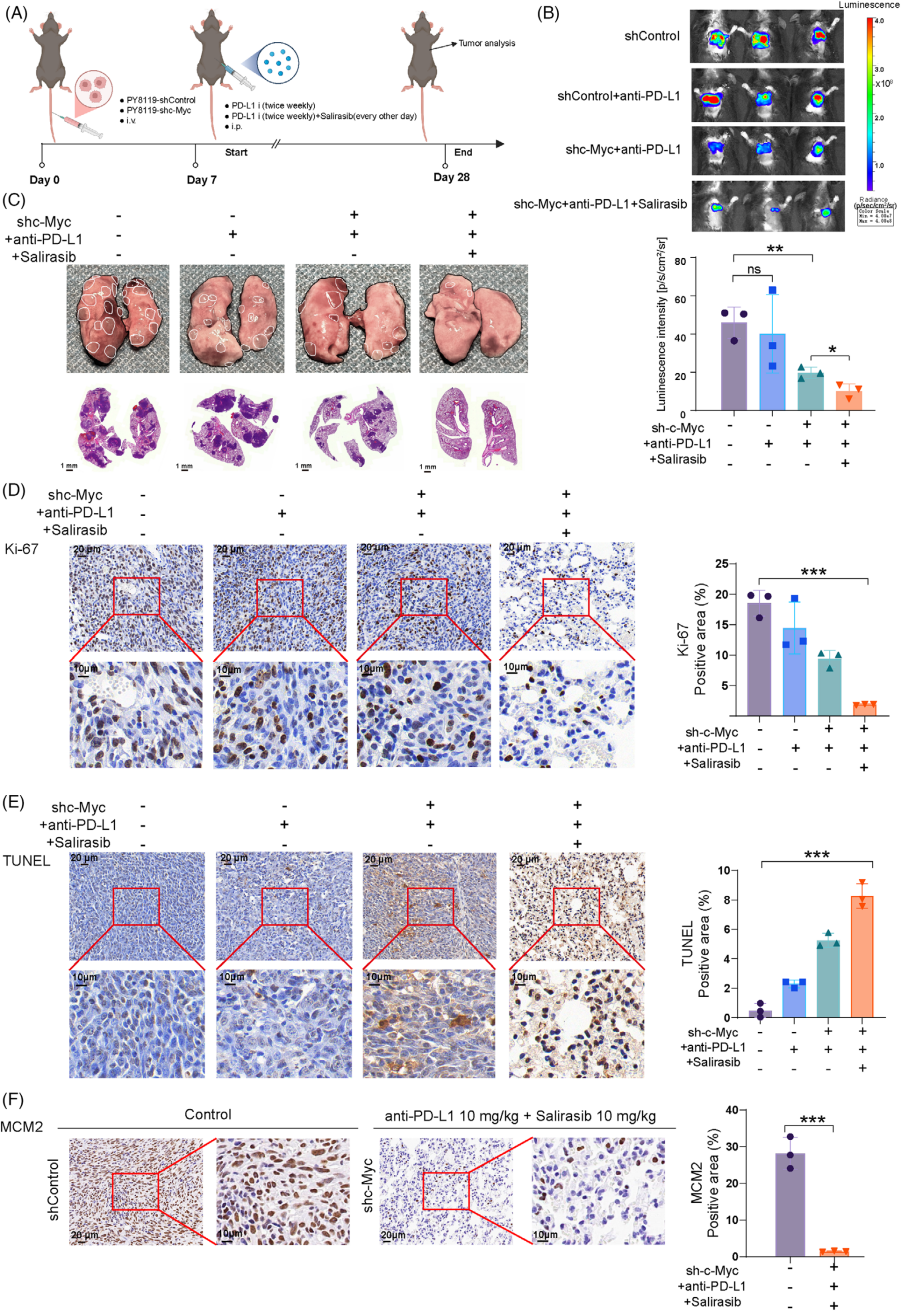
Targeting c‐Myc synergises with Salirasib significantly enhanced the anti‐TNBC effect of immunotherapy in murine lung‐metastatic TNBC model. (A) Timeline of establishment and treatment of murine models of lung‐metastatic TNBC. (B, C) Bioluminescence imaging and HE staining revealed that targeting c‐Myc combined with Salirasib synergistically inhibited lung metastasis of circulating Py8119 cells. (D, E) Immunohistochemical analysis of Ki‐67 and TUNEL indicated that targeting c‐Myc combined with Salirasib significantly enhanced the antitumour effect of immunotherapy in metastatic lung tissues. (F) Immunohistochemical staining revealed a reduced expression of MCM2 in the shc‐Myc group treated with the PD‐L1 inhibitor and Salirasib compared to the shControl group. Data were summarised as means  ±  SD. *p  < .05; **p  < .01; ***p  < .001.

We evaluated the therapeutic efficacy of combining c‐Myc inhibition with Salirasib in metastatic TNBC. Breast cancer lung metastasis models were generated in mice through tail vein injection of Py8119‐shControl or Py8119‐shc‐Myc cells. Mice were divided into four groups: shControl, shControl with PD‐L1 inhibitor treatment, shc‐Myc with PD‐L1 inhibitor treatment, and shc‐Myc with both PD‐L1 inhibitor and Salirasib treatment (Figure 9A). To assess the metastatic capacity of Py8119‐luc cells in vivo, bioluminescence imaging was employed. When compared to the shControl group, both groups treated with the PD‐L1 inhibitor showed decreased metastatic load, evident from the reduced region of interest (ROI) values in their lungs. Importantly, the shc‐Myc group that received both the PD‐L1 inhibitor and Salirasib exhibited the most significant decrease in lung metastases (Figure 9B). Following this, lung tissues were collected for histopathological assessment, and H&E staining was performed to identify metastatic lesions (Figure 9C). The lung metastasis model showed that the shc‐Myc group receiving both the PD‐L1 inhibitor and Salirasib exhibited marked inhibition of circulating TNBC cell metastasis. IHC analysis further revealed that this combination treatment resulted in the most pronounced suppression of Ki‐67 and the most notable upregulation of TUNEL, indicating decreased proliferation and increased apoptosis, respectively (Figure 9D and E). Additionally, we assessed MCM2 expression in the lungs of mice from the shControl group and the shc‐Myc group treated with both the PD‐L1 inhibitor and Salirasib. Notably, MCM2 expression were markedly decreased in the latter group, showing substantial downregulation relative to the shControl group (Figure 9F).

Given the established efficacy of the novel combined therapy involving the PD‐L1 inhibitor in TNBC, we subsequently conducted a safety evaluation of this treatment regimen. Mice were administered intraperitoneal injections of Salirasib on alternate days (specifically, Days 1, 3, 5, and 7) and the PD‐L1 inhibitor twice weekly (on Days 3 and 6). During the course of the experiment, minor variations in body weight were noted among mice across different intervention groups, albeit without statistical significance (Figure S5A). Whole blood samples (*n* = 3) were collected from the orbital venous plexus 24 h post‐final treatment, and serum was then extracted via centrifugation for clinical biochemical analyses. Additionally, major organs such as the heart and kidneys were excised to perform histopathological examination using HE staining. In comparison to untreated shControl group, the shc‐Myc group receiving the combined Salirasib and PD‐L1 inhibitor treatment showed no notable changes in serum levels of several major biochemical indicators such as aspartate aminotransferase (AST) and alanine aminotransferase (ALT) (Figure S5B–E). Furthermore, histological assessments revealed no apparent pathological abnormalities in the examined organs after the combined therapy (Figure S5F). Taken together, these biochemical and histopathological findings indicate that the concurrent administration of Salirasib and the PD‐L1 inhibitor exhibits minimal toxicity, thus supporting its safety for combined therapeutic use. The lung metastasis model revealed that the combined treatment notably hindered the metastasis of tumour cells. In conclusion, Salirasib stands out as a promising small‐molecule candidate for TNBC therapy, exhibiting potent antitumour activity and markedly enhancing immune checkpoint blockade efficacy through its synergism with c‐Myc targeting.

## 4 DISCUSSION

**FIGURE 10:**
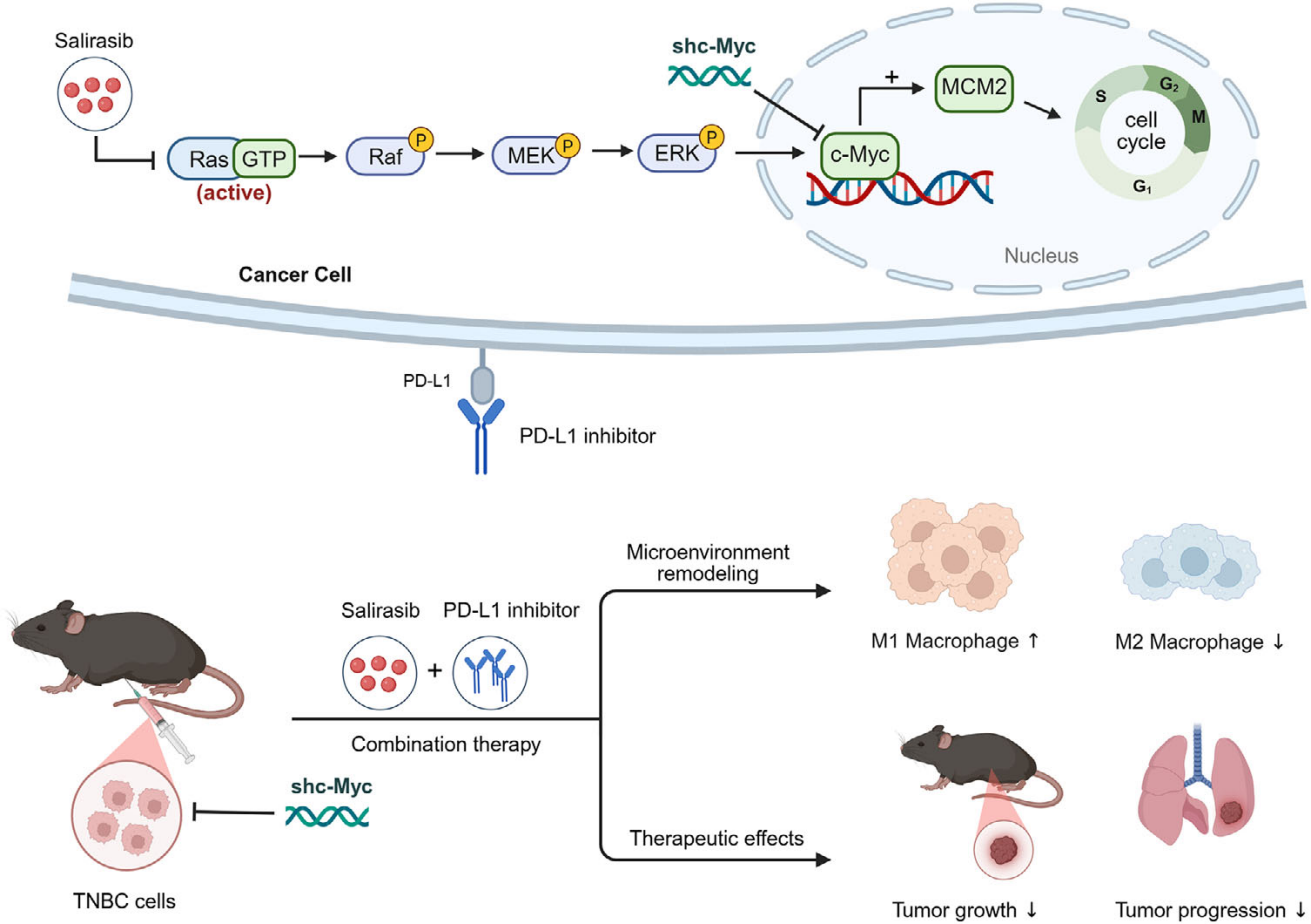
Overall mechanism diagram of the enhanced immunotherapy efficacy with the synergism of targeting c‐Myc and the Ras inhibitor Salirasib.

TNBC, a clinically aggressive breast cancer category, is notable for its absence of targeted therapies and poor prognosis.^35^ Merely a portion of individuals with TNBC derive benefit from ICIs, and complete, durable remissions are rare. This underscores the urgent need for therapeutic targets that can enhance the efficacy of immunotherapy. Aberrant c‐Myc activation is frequently observed in TNBC, and elevated c‐Myc expression has been linked to resistance to ICI therapy.^36^ Previous studies, including our own, demonstrated that c‐Myc suppression can improve the therapeutic effects of immunotherapy for TNBC,^12^, ^36^, ^37^, ^38^ and lead to improved immune cell infiltration observed after c‐Myc inhibition.^39^ c‐Myc is crucial for modulating cell‐to‐cell communication in TME, and its bidirectional interactions with elements of the TME facilitate TNBC progression.^11^ Targeting c‐Myc rejuvenates TME and promotes the recruitment of immune cells, collectively fostering conditions favourable for immunotherapy. Conversely, PD‐L1 activity enhances the function of c‐Myc, which supports tumour cell growth and promotes immune escape.^40^ Furthermore, our prior research has indicated that remodelling of the c‐Myc pathway alters tumour cell cycle progression^8^, ^15^, ^41^ and stemness,^9^, ^10^ thereby modulating tumour cell sensitivity to CDK inhibitors. These findings suggest that c‐Myc represents a promising therapeutic target for TNBC, and cell cycle regulation may be a key mechanism by which c‐Myc impacts the malignant phenotype of TNBC cells. Considering these insights, we suggest a therapeutic approach which integrates targeting c‐Myc with agents targeting the cell cycle to achieve synergistic antitumour effects and improved immunotherapy responsiveness in TNBC. (See Figure 10)

Multiple studies have been conducted to screen functional drug libraries targeting various cancer cell pathways to discover potential anticancer agents.^13^, ^42^ Despite the challenges posed by c‐Myc, an intrinsically disordered oncoprotein without druggable pockets, we have previously explored unconventional strategies to achieve its inhibition.^12^, ^14^ In this study, we shifted our focus from solely targeting c‐Myc to examining its synergistic effects with other potential agents, aiming to enhance antitumour efficacy and expand therapeutic options. Initially, we established c‐Myc knockdown TNBC cell models to identify compounds that demonstrate synergism with c‐Myc inhibition. Next, we validated this synergy by evaluating changes in cell proliferation, migration, invasion, and apoptosis. Furthermore, we developed murine TNBC models for both primary and metastatic tumours to assess the combined antitumour effect and potential immunotherapy enhancement in vivo. Considering that TME remodelling plays a pivotal role in tumour progression, we investigated whether the combined therapy influenced the polarisation state of M1/M2 macrophages. To accomplish these objectives, we tackled three primary challenges: identifying synergistic anticancer agents when combined with c‐Myc targeting, validating the antitumour and immunotherapy‐boosting effects in primary and metastatic TNBC models, and elucidating the cellular mechanisms behind the synergy between c‐Myc inhibition and specific small‐molecule agents.

In this study, we have uncovered significant synergistic effects between the Ras inhibitor Salirasib and c‐Myc targeting in TNBC cells. Specifically, this combination effectively inhibits cell migration and invasion while enhancing cell apoptosis. Ras proteins, belonging to the small GTPase family, play an essential role in cellular signalling networks that control cell growth, proliferation, and survival.^30^ The activation of these proteins triggers multiple downstream pathways, ultimately causing the activation of c‐Myc target genes. These genes subsequently drive critical cancer hallmarks, including metastasis, invasion, altered metabolism, and immune evasion. Previous studies have shed light on the cooperative interplay between Ras and c‐Myc in cell‐intrinsic mechanisms underlying tumourigenesis.^43^, ^44^ The inhibition of Ras has shown promise in potentiating chemotherapeutic and molecularly targeted agents.^29^ Additionally, TNBC patients with high expression of KRAS, the most common oncogenic isoform of the Ras family, exhibit significantly better survival rates compared to those with low KRAS expression (Figure S2B). Therefore, targeting Ras using Salirasib in TNBC represents a compelling and potential strategy for synergising with c‐Myc targeting. Kortlever et al. pointed out that c‐Myc‐Ras cooperation induces immune suppression by reprogramming inflammatory pathways,^24^ which aligns with our finding that simultaneously inhibiting c‐Myc and Ras alters the TME by polarising macrophages toward an antitumoural M1 phenotype. Regarding the enhancement of immunotherapy efficacy, the repolarisation of macrophages from M2 to M1 observed in murine model of TNBC reversed immunosuppressive TME and potentiated better therapeutic outcomes. Further, mechanisms and applications in more humanised models, however, remain to be clarified in subsequent studies.

The Raf‐MEK‐ERK cascade constitutes a major MAPK pathway and is among the most thoroughly characterised downstream effectors of Ras signalling. After activation of Ras, Raf kinase is translocated to the plasma membrane and becomes activated, initiating a phosphorylation cascade through MEK1/2 and ERK1/2.^45^, ^46^, ^47^ Activated ERK1/2 subsequently transits into the nucleus to phosphorylate diverse nuclear effectors including c‐Myc, thereby promoting cellular proliferation, differentiation, survival, and invasion.^48^, ^49^, ^50^ Salirasib exerts potent antitumour effects by disrupting Ras‐mediated signalling cascades, thus inhibiting cell cycle progression, attenuating proliferation, and suppressing tumour growth and metastasis.^51^ Identifying the specific Ras downstream pathways predominantly activated in distinct tumours could enhance the efficacy of targeted therapeutic strategies. In our study, RNA‐seq analysis revealed that the MAPK signalling pathway was enriched among the downregulated DEGs (Figure 5D), consistent with KEGG results of TNBC patients with dual‐low MYC and KRAS expression from TCGA database (Figure S2C). Furthermore, KEGG enrichment analysis also highlighted the downregulated DEGs enriched in cell cycle and DNA replication pathways. From the intersecting genes within the top 50 DEGs of both TNBC cell lines, we specifically screened out MCM2, a crucial regulator of DNA replication initiation. DNA replication is essential for accurate genome duplication and significantly influences the onset and development of TNBC. Previous researches have indicated that elevated expression of the MCM protein family correlates strongly with poorer survival outcomes among patients with breast cancer.^52^ MCM2 was found to be highly expressed in TNBC patient with dual‐high MYC and KRAS expression from the TCGA database (Figure 5F), with lower expression levels correlating to improved overall survival (Figure 5H). Correspondingly, we confirmed lower MCM2 expression levels in murine models treated with combined therapy compared to controls, in both primary and metastatic tumour lesions. From these results, we speculated that activation of the MAPK pathway and elevated MCM2 expression may drive the aggressive phenotype of c‐Myc‐positive TNBC, whereas co‐targeting c‐Myc and Ras signalling with Salirasib may suppress tumour progression.

Generally speaking, our findings suggest that combined c‐Myc inhibition and Ras inhibitor holds translational potential by enhancing immunotherapy efficacy in TNBC. Nevertheless, several limitations should be acknowledged. Firstly, the ICI efficacy was validated only in a single syngeneic murine model. In addition, c‐Myc targeting was achieved by shRNA knockdown, whereas clinically feasible modalities including targeted degraders or nucleic acid‐based strategies remain to be evaluated for pharmacokinetics and safety. Also, the immune profiling was limited to macrophage markers by mIF and higher‐resolution techniques, including flow cytometry and single‐cell RNA sequencing, are warranted to comprehensively delineate immune cell subsets. Future studies addressing these issues will be essential to advance this synergistic enhancement toward clinical translation.

## 5 CONCLUSIONS

In summary, our study identified a synergistic antitumour effect by combining c‐Myc inhibition with Salirasib in c‐Myc–overexpressing TNBC cells, using a drug library screening approach. This combination significantly enhanced both antitumour activity and immunotherapy efficacy. In vivo validation in primary and metastatic TNBC models further confirmed the therapeutic potential. These findings provide evidence for a potential combination approach to enhance immunotherapy efficacy in c‐Myc–positive TNBC.

## AUTHOR CONTRIBUTIONS

X.X. Guan and Y.Q. Shi contributed to the conception of the idea and design of the study. X.J. Liang, F.Y. Gao, and Y. Zhu contributed to the conduction of the experiments. X.J. Liang, Y.Q. Liu, and Y.H. Zhao contributed to the analysis of the raw data. Y. Zhu and F. Ye contributed to administrative and technical support. X.J. Liang and Y.Q. Liu contributed to writing and revising the manuscript. X.X. Guan contributed to supervising the study and reviewing the manuscript. All authors read and approved the final manuscript.

## CONFLICT OF INTEREST STATEMENT

The authors have no conflicts of interest to declare.

## FUNDING

This study was supported by grants from the National Natural Science Foundation of China (No. 82303622 and No. 82430093).

## ETHICS STATEMENT

This study was conducted in strict accordance with established ethical guidelines for animal research. All animal experimental procedures were reviewed and approved by the Institutional Animal Care and Use Committee (IACUC) of Nanjing Medical University (Approval No. IACUC‐2409020) and performed following the guidelines outlined in the Guide for the Care and Use of Laboratory Animals.

## CONSENT FOR PUBLICATION

All authors have approved the manuscript for publication. As the manuscript does not contain individual personal data, further consent for publication is unnecessary.

## Supporting information



Supporting Information

## Data Availability

All data generated in this study are presented in the main text and supplementary materials. Additional information and requests for resources should be directed to the lead contact, Xiaoxiang Guan (xguan@njmu.edu.cn), who will fulfil reasonable requests.
