# Supplementary material for: Targeting c‐Myc enhances immunotherapy efficacy in combination with Ras inhibitor in triple‐negative breast cancer
Source: Clin Transl Med. 2025 Sep 29;15(10):e70484. doi: 10.1002/ctm2.70484 (PMC12477544; doi:10.1002/ctm2.70484)
Supplement: Supplementary file 1 — Supporting Information [file CTM2-15-e70484-s001.docx]

**Supplementary Figures**

**Targeting c-Myc enhances immunotherapy efficacy in combination with Ras inhibitor in triple-negative breast cancer**

Xiaojie Liang^1†^, Yiqiu Liu^1†^, Ye Zhu^1†^, Yuhan Zhao^1^, Fan Ye^1^, Fangyan Gao^1^, Yaqin Shi^3*†^, Xiaoxiang Guan^1,2*†^


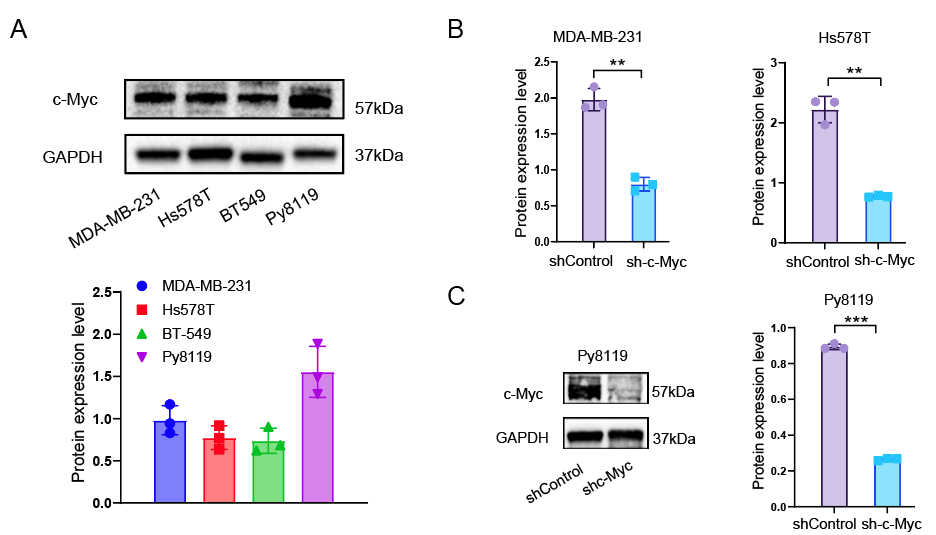


**Supplementary Fig.1. c-Myc expression across TNBC cell lines and its efficient depletion by shRNA**

1. Endogenous c-Myc protein levels in 4 TNBC cell lines (human MDA-MB-231, Hs578T, BT-549 and murine Py8119) were assessed by Western blot (top) and normalized to GAPDH (bottom).
2. Stable shRNA-mediated knockdown of c-Myc in MDA-MB-231 (left) and Hs578T (right) cells markedly reduces protein expression relative to shControl. Data represent mean ± SD from three independent experiments; *p* < 0.01 (two‐tailed Student’s t-test).
3. Efficient c-Myc depletion in Py8119 cells (a murine TNBC line derived from C57BL/6 mice) was shown by Western blot (left) and quantification (right). Data represent mean ± SD (n = 3); *p* < 0.001 (two‐tailed Student’s t-test).


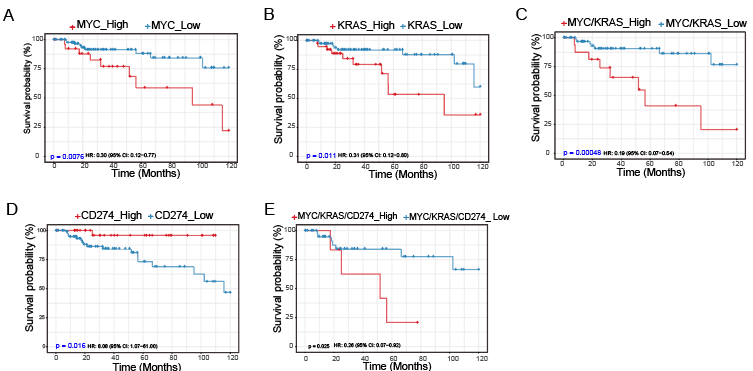


**Supplementary Fig.2. MYC expression was related to prognosis in TNBC patients based on the TCGA database.**

1. High MYC expression was associated with significantly poorer survival compared with low MYC expression.
2. High KRAS expression predicted worse outcome relative to low KRAS.
3. Concurrent high MYC and KRAS marked an even more unfavorable prognosis than either alone.
4. Elevated CD274 (PD-L1) correlated with improved survival among TNBC patients.
5. Patients exhibiting high expression of MYC, KRAS and CD274 (PD-L1) together showed the most favorable overall survival.


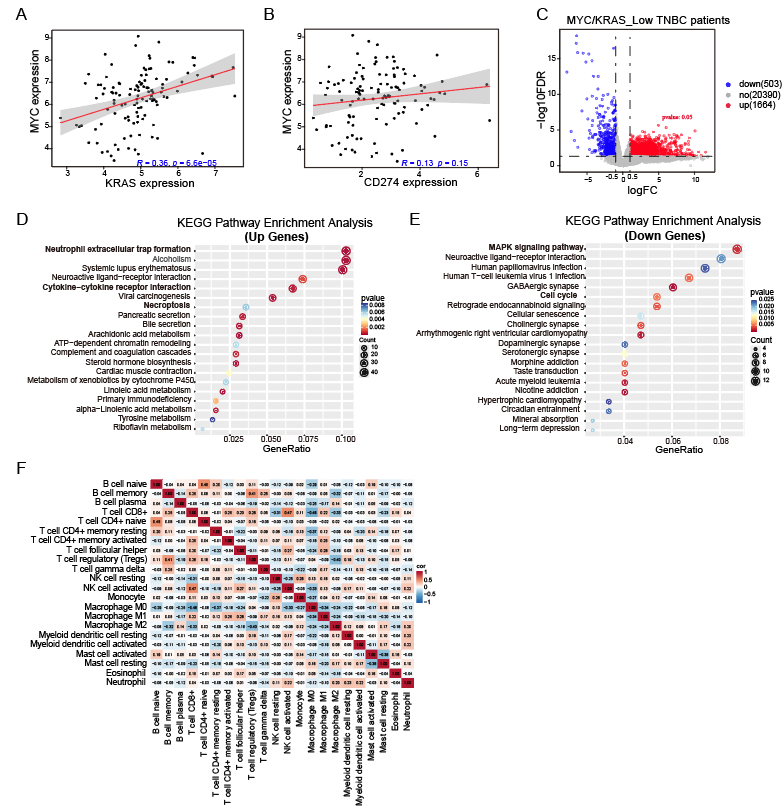


**Supplementary Fig.3. MYC expression was related to prognosis in TNBC patients based on the TCGA database.**

(A) and (B) Relationship between MYC and PD-L1 and KRAS expressions in TNBC from the TCGA database.

(C) The volcano plot of DEGs in TNBC patients with dual low expression of MYC and KRAS from the TCGA database.

(D) and (E) KEGG pathway enrichment of analysis of up and down DEGs of (H).

(F) Correlation heat map of 22 types of immune cells. Positive correlation was shown in RED, and negative correlation was shown in BLUE.

**
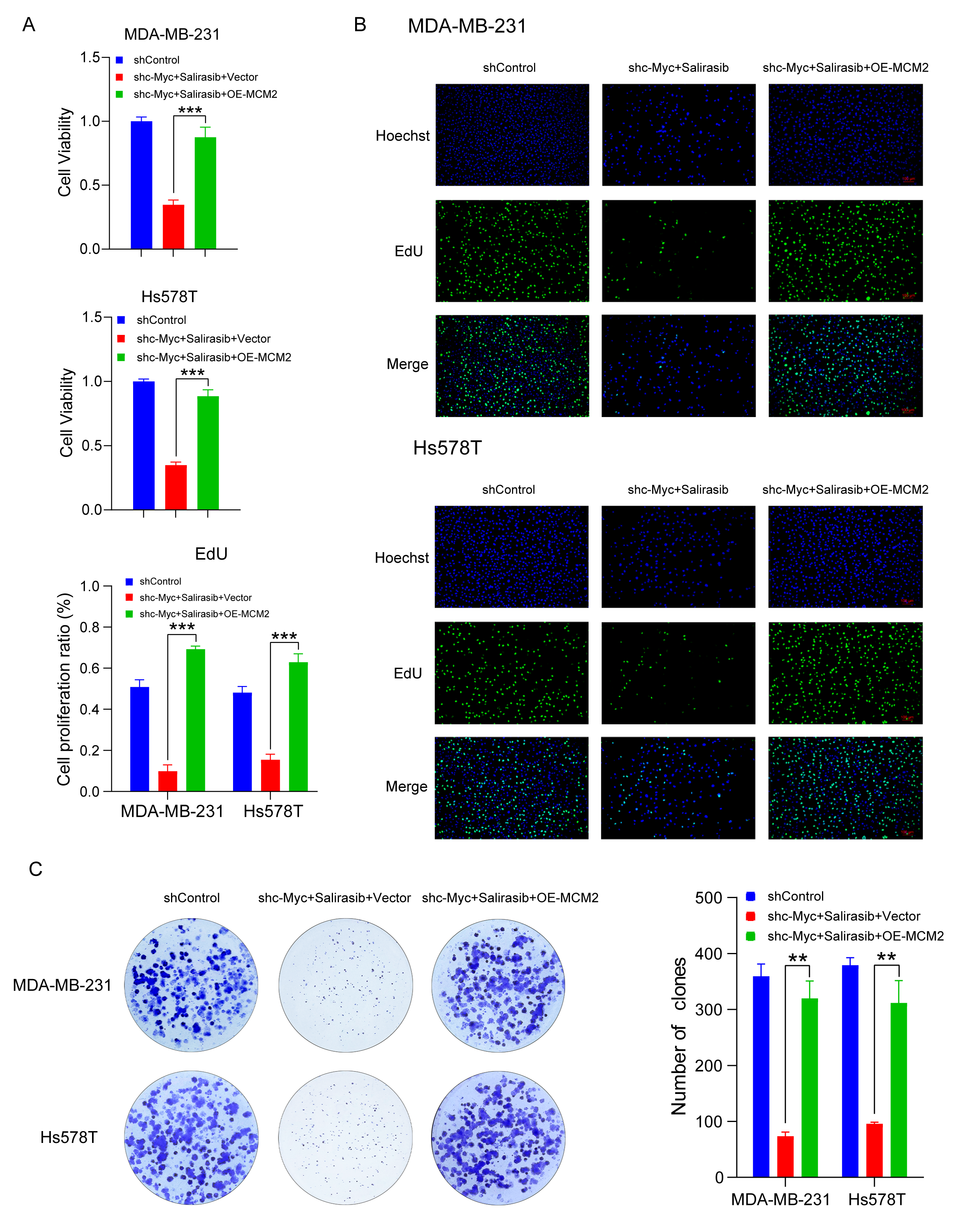
**

**Supplementary Fig.4. Characterization of overexpressing MCM2 on the combined use of Salirasib with targeted inhibition of c-Myc for rescue experiments.**

1. CCK-8 assays were used to assess the viability of MDA-MB-231 and Hs578T cell lines following MCM2 overexpression rescue.
2. EdU cell proliferation assays were utilized to examine the cell viability of MDA-MB-231 and Hs578T cells following the rescue treatment.

(C) Colony formation assay were applied to exam long-term cell survival after rescue treatment.

Data were summarized as means ± SD. **P* < 0.05; ***P* < 0.01; ****P* < 0.001


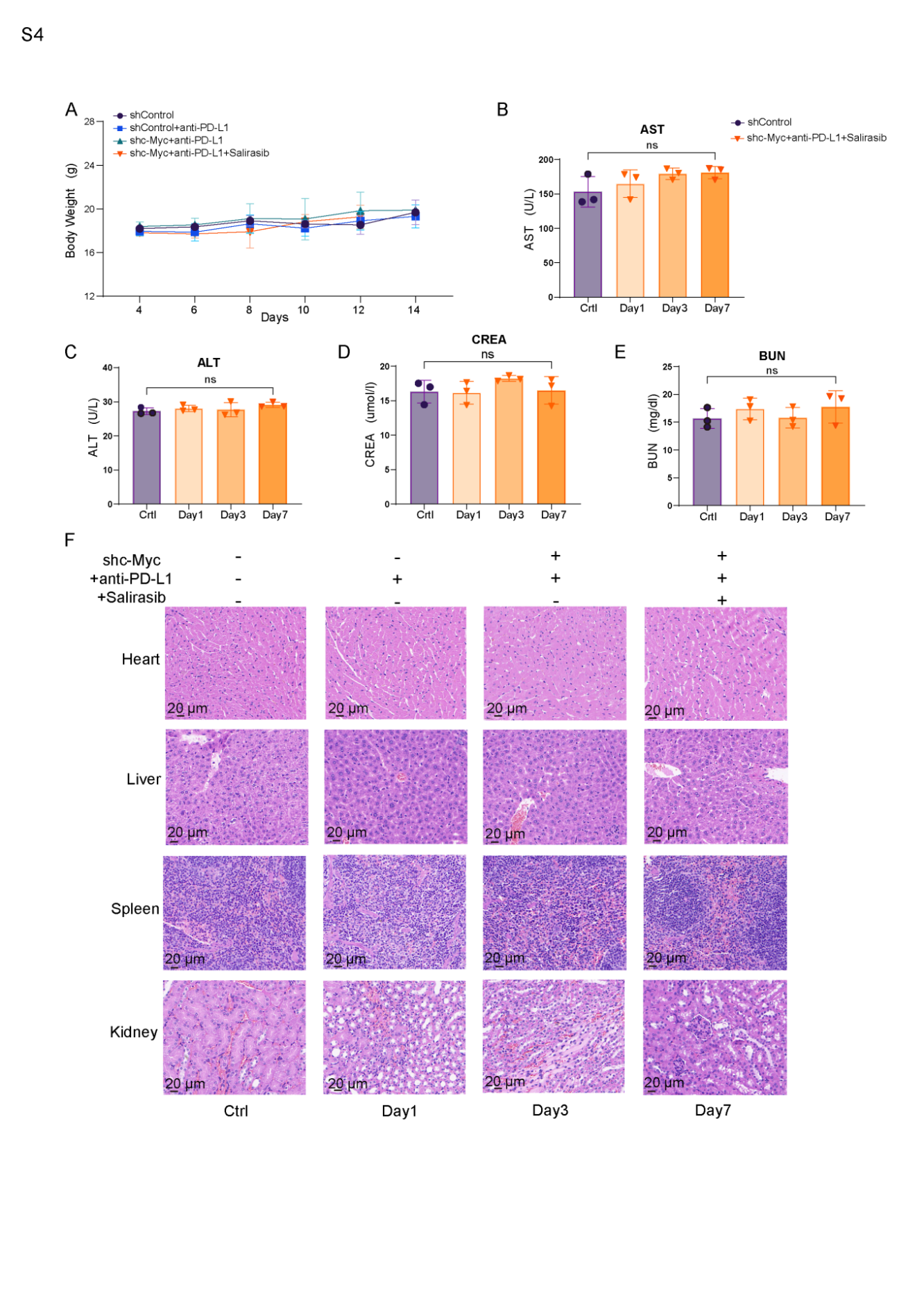


**Supplementary Fig.5. Safety evaluation on the synergy of targeting c-Myc and Salirasib**

1. Alterations in murine body weight of each treatment group.

(B)-(E) Biochemical tests on serum AST, ALT, CEA and BUN levels of mice after combined treatment on day 1, day 3 and day 7 (ns, no significance).

(F) Histological structures of main organs ( heart, liver, spleen, and kidneys) exhibited no significant changes after combined treatment on day 1, day 3 and day 7 (scale bar= 20 μm). (ns, no significance).

Data were summarized as means ± SD.

**Supplementary Table 1. Primer information required for RT-qPCR process**

| Gene name | Forward primer sequence (5’-3’) | Reverse primer sequence (5’-3’) |
| --- | --- | --- |
| GAPDH | TGTTGCCATCAATGACCCCTT | CTCCACGACGTACTCAGCG |
| c-Myc | GGACTTGTTGCGGAAACGAC | CTCAGCCAAGGTTGTGAGGT |
| MCM2 | TGCCAGCATTGCTCCTTCCATC | AAACTGCGACTTCGCTGTGCCA |
| MCM7 | GCCAAGTCTCAGCTCCTGTCAT | CCTCTAAGGTCAGTTCTCCACTC |
| DEK | TGGGTCAGTTCAGTGGCTTTCC | CTCTCCAAATCAAGAACCTCACAG |
| RUVBL1 | GAAGACAGAGGTGCTGATGGAG | CTCTGTCTCACACGGAGTTAGC |
| KIF14 | GCACTTTCGGAACAAGCAAACCA | ATGTTGCTGGCAGCGGGACTAA |
| WDR43 | CCTCCACAAACCGAGCAAGTAG | GCTATTCGTCTGGAGGTCTTCC |
| IGFBP13 | CGCTACAAAGTTGACTACGAGTC | GTCTTCCATTTCTCTACGGCAGG |
| FBXO32 | CACTGGTCCAAAGAGTCGGCAA | GCACAAAGGCAGGTCAGTGAAG |
| PLEKHA6 | CAGTGAGGACATCTATGCTGACC | ACTTGTAGGTGTGGAGGCTGTC |

**Supplementary Table S2. Antibodies information required for Western Blot experiment process**

| Antibody name | Manufacturer | Catalog No. |
| --- | --- | --- |
| anti-BCL2 | Proteintech | 80313-1-RR |
| anti-BAX | Proteintech | 60267-1-Ig |
| anti-E-cadherin | Proteintech | 80541-5-RR |
| anti-MMP9 | Proteintech | 27306-1-AP |
| anti-Vimentin | Proteintech | 80232-1-RR |
| anti-MMP2 | ABclonal | A19080 |
| anti-Ras | ABclonal | A19779 |
| anti-Raf | ABclonal | A15033 |
| anti-p-Raf | ABclonal | AP0012 |
| anti-MEK1/MEK2 | ABclonal | A4868 |
| anti-p-MEK1/MEK2 | ABclonal | AP1349 |
| anti-ERK1/2 | ABclonal | A4782 |
| anti-p-ERK1/2 | ABclonal | AP0974 |
| anti-c-Myc | Cell Signaling Technology | #5605 |
| anti-CyclinD1 | Cell Signaling Technology | #2978 |
| anti-CDK4 | Cell Signaling Technology | #12790 |
| anti-CDK6 | Cell Signaling Technology | #13331 |
| anti-Rb | Cell Signaling Technology | #9313 |
| anti-p-Rb | Cell Signaling Technology | #9301 |
| anti-MCM2 | Cell Signaling Technology | #3619 |
| anti-GAPDH | Beyotime | AF2823 |
| anti-c-Myc | HUABIO | HA721182 |

**Supplementary Table S3. shRNA sequences**

| shRNAs | Sequence (5’-3’) |
| --- | --- |
| c-Myc shRNA1 | GGAAACGACGAGAACAGTTGA |
| c-Myc shRNA2 | GCTCATTTCTGAAGAGGACTT |
| shControl | UUCUCCGAACGUGUCACGUTT |
| Myc-Mus-925 | TGGAGATGATGACCGAGTTAC |
| Myc-Mus-1011 | ATCATCATCCAGGACTGTATG |

**Supplementary Table S4. ChIP primer sequences**

|  | Sequence (5’-3’) |
| --- | --- |
| Forward | GTTCTTAGGGAGGGGCTTGC |
| Reverse | TTATCTTTGCCCAGGGGAGC |

**Supplementary Table S5. Abbreviations**

| TNBC | Triple-negative breast cancer |
| --- | --- |
| MCM2 | Maintenance Complex Component 2 |
| TME | Tumor microenvironment |
| TIME | Tumor immune microenvironment |
| ER | estrogen receptor |
| PR | progesterone receptor |
| HER2 | Human Epidermal Growth Factor Receptor 2 |
| ORR | overall response rate |
| ICIs | immune checkpoint inhibitors |
| XNA | xenic nucleic acids |
| CDK | cyclin-dependent kinase |
| EMT | epithelial-to-mesenchymal transition |
| CCK-8 | Cell counting kit 8 |
| EdU | 5-Ethynyl-2′-deoxyuridine |
| RT-qPCR | Real-time quantitative PCR |
| PI | propidium iodide |
| H&E | Hematoxylin and eosin |
| IF | immunofluorescence |
| IHC | immunohistochemical |
| TCGA | The Cancer Genome Atlas |
| BRCA | breast invasive carcinoma |
| KEGG | Kyoto Encyclopedia of Genes and Genomes |
| DEGs | differentially expressed genes |
| MAPK | mitogen-avtivated protein kinase |
| TIIC | tumor-infiltrating immune cell |
| TAM | tumor-associated macrophage |
| AST | aspartate aminotransferase |
| ALT | alanine aminotransferase |
| CREA | creatinine |
| BUN | blood urea nitrogen |
| ROI | region of interest |
| IACUC | the Institutional Animal Care and Use Committee |
